# Supplementary material for: Drivers of rodent community structure in an Urban National Park, Kenya
Source: PLoS One. 2025 Apr 16;20(4):e0321659. doi: 10.1371/journal.pone.0321659 (PMC12002537; doi:10.1371/journal.pone.0321659)
Supplement: S1 Table — (DOCX) [file pone.0321659.s001.docx]

# S1: Model selection table for species abundance

# S2: Model selection table for rodent species richness

# S3: Model selection table for Shannon Weiner diversity Index

# S4: Model selection table Brillouin index

# S5: Model selection table for Simpson’s diversity Indexs
