# Supplementary material for: Drivers of rodent community structure in an Urban National Park, Kenya
Source: PLoS One. 2025 Apr 16;20(4):e0321659. doi: 10.1371/journal.pone.0321659 (PMC12002537; doi:10.1371/journal.pone.0321659)
Supplement: S3 Table — (DOCX) [file pone.0321659.s003.docx]

**S3_Table. Model selection table for Shannon Weiner diversity Index**
