# Supplementary material for: Drivers of rodent community structure in an Urban National Park, Kenya
Source: PLoS One. 2025 Apr 16;20(4):e0321659. doi: 10.1371/journal.pone.0321659 (PMC12002537; doi:10.1371/journal.pone.0321659)
Supplement: Attachment — Submitted filename: PONE-D-23-23260.pdf [file pone.0321659.s006.pdf]

PLOS ONE

# Drivers of Rodent Community Structure in an Urban National Park, Kenya

--Manuscript Draft--

| | |
|---|---|
| **Manuscript Number:** | PONE-D-23-23260 |
| **Article Type:** | Research Article |
| **Full Title:** | Drivers of Rodent Community Structure in an Urban National Park, Kenya |
| **Short Title:** | Rodent Community Structure in an Urban National Park, Kenya |
| **Corresponding Author:** | Olivia Wesula Lwande, PhD<br>Umea Universitet<br>Umea, VASTERBOTTENS LAN SWEDEN |
| **Keywords:** | |
| **Abstract:** | Nairobi National Park's ecosystem is among Kenya's most vulnerable because of the extreme pressure of urbanization. Understanding the structure of the rodent community in Nairobi National Park is important because rodents are sensitive bioindicators of ecosystem health. The association of vegetation type, vegetation metrics, seasonality, and anthropogenic disturbance with rodent community structure was also evaluated. To achieve this,  rodent samples were collected from 15 sites during the wet and dry seasons to assess rodent diversity and relative abundance. Capture-release method was used to trap rodents in the savannah, forest, and riverine vegetation types of Nairobi National Park during the dry and wet seasons. From 56 rodents trapped, five species identified were Lemniscomys striatus, Hylomyscus sp, Rattus rattus, Mus mus and Otomys tropicalis. Rodent diversity at NNP was low (Simpson=0.7130; Shannon Weiner=1.40; Brillouin index=1.27) while Pielou's species evenness, was moderate=0.44 indicating near equity in species distribution. Univariate Generalised linear models  showed that rodent abundance was influenced by season, vegetation type, and vegetation metrics but not by human disturbance/edge effects. The multivariate model indicated that rodents were abundant in the wet season compared to the dry season, and that abundance was also positively associated with increased tree and shrub densities. Rodent species richness was positively associated with higher tree density, while vegetation types influenced rodent species diversity. Rodent abundance was influenced by vegetation type, vegetation metrics (density and cover), and season but not by human disturbance. It was observed that the diverse anthropogenic activities occurring in NNP, do not significantly influence rodent abundance as compared to the biotic and abiotic factors. Factors that influence vegetation and season, like climate change, are thus a threat to the faunal diversity in NNP. This first rodent survey in this Park,  provide preliminary data for continued monitoring of this ecosystem. |
| **Order of Authors:** | Immaculate M. Mungai, Master |
| | Nathan Gichuki |
| | Dorcus A.O. Sigana |
| | Benard Agwanda |
| | Patrick Chiyo |
| | Vincent Obanda |
| | Olivia Wesula Lwande, PhD |
| **Additional Information:** | |
| **Question** | **Response** |
| **Financial Disclosure**<br><br>Enter a financial disclosure statement that describes the sources of funding for the work included in this submission. Review | Initial of author who received the award> OWL<br>The study was supported by the Swedish Research Council, Research network grant with a focus on Swedish research links, Registration number 2021-05307.<br><br>URL: https://www.vr.se/english.html |

| | |
|---|---|
| the submission guidelines for detailed requirements. View published research articles from *PLOS ONE* for specific examples.<br><br>This statement is required for submission and **will appear in the published article** if the submission is accepted. Please make sure it is accurate.<br><br>**Unfunded studies**<br>Enter: *The author(s) received no specific funding for this work.*<br><br>**Funded studies**<br>Enter a statement with the following details:<br>• Initials of the authors who received each award<br>• Grant numbers awarded to each author<br>• The full name of each funder<br>• URL of each funder website<br>• Did the sponsors or funders play any role in the study design, data collection and analysis, decision to publish, or preparation of the manuscript?<br>• **NO** - Include this sentence at the end of your statement: *The funders had no role in study design, data collection and analysis, decision to publish, or preparation of the manuscript.*<br>• **YES** - Specify the role(s) played.<br><br>* typeset | The funders had no role in study design, data collection and analysis, decision to publish, or preparation of the manuscript. |
| **Competing Interests**<br><br>Use the instructions below to enter a competing interest statement for this submission. On behalf of all authors, disclose any competing interests that could be perceived to bias this work—acknowledging all financial support and any other relevant financial or non-financial competing interests.<br><br>This statement is required for submission and **will appear in the published article** if the submission is accepted. Please make sure it is accurate and that any funding sources listed in your Funding Information later in the submission form are also | The authors have declared that no competing interests exist. |

Powered by Editorial Manager® and ProduXion Manager® from Aries Systems Corporation

| | |
|---|---|
| declared in your Financial Disclosure statement.<br><br>View published research articles from *PLOS ONE* for specific examples.<br><br>**NO authors have competing interests**<br><br>Enter: *The authors have declared that no competing interests exist.*<br><br>**Authors with competing interests**<br><br>Enter competing interest details beginning with this statement:<br><br>*I have read the journal's policy and the authors of this manuscript have the following competing interests: [insert competing interests here]*<br><br>* typeset | |
| **Ethics Statement**<br><br>Enter an ethics statement for this submission. This statement is required if the study involved:<br><br>• Human participants<br>• Human specimens or tissue<br>• Vertebrate animals or cephalopods<br>• Vertebrate embryos or tissues<br>• Field research<br><br>Write "N/A" if the submission does not require an ethics statement.<br><br>General guidance is provided below. Consult the submission guidelines for detailed instructions. **Make sure that all information entered here is included in the Methods section of the manuscript.** | The study was approved by Kenya Wildlife Service, permit number KWS/BRP/5001. The study was non-destructive because used non-lethal traps under the capture and release method. |

Powered by Editorial Manager® and ProduXion Manager® from Aries Systems Corporation

| | |
|---|---|
| **Format for specific study types**<br><br>**Human Subject Research (involving human participants and/or tissue)**<br>• Give the name of the institutional review board or ethics committee that approved the study<br>• Include the approval number and/or a statement indicating approval of this research<br>• Indicate the form of consent obtained (written/oral) or the reason that consent was not obtained (e.g. the data were analyzed anonymously)<br><br>**Animal Research (involving vertebrate animals, embryos or tissues)**<br>• Provide the name of the Institutional Animal Care and Use Committee (IACUC) or other relevant ethics board that reviewed the study protocol, and indicate whether they approved this research or granted a formal waiver of ethical approval<br>• Include an approval number if one was obtained<br>• If the study involved *non-human primates*, add *additional details* about animal welfare and steps taken to ameliorate suffering<br>• If anesthesia, euthanasia, or any kind of animal sacrifice is part of the study, include briefly which substances and/or methods were applied<br><br>**Field Research**<br><br>Include the following details if this study involves the collection of plant, animal, or other materials from a natural setting:<br>• Field permit number<br>• Name of the institution or relevant body that granted permission | |
| **Data Availability**<br><br>Authors are required to make all data underlying the findings described fully available, without restriction, and from the time of publication. PLOS allows rare exceptions to address legal and ethical concerns. See the PLOS Data Policy and FAQ for detailed information. | Yes - all data are fully available without restriction |

| | |
|---|---|
| A Data Availability Statement describing where the data can be found is required at submission. Your answers to this question constitute the Data Availability Statement and **will be published in the article**, if accepted.<br><br>**Important:** Stating 'data available on request from the author' is not sufficient. If your data are only available upon request, select 'No' for the first question and explain your exceptional situation in the text box.<br><br>Do the authors confirm that all data underlying the findings described in their manuscript are fully available without restriction? | |
| **Describe where the data may be found in full sentences. If you are copying our sample text, replace any instances of XXX with the appropriate details.**<br><br>• If the data are **held or will be held in a public repository**, include URLs, accession numbers or DOIs. If this information will only be available after acceptance, indicate this by ticking the box below. For example: *All XXX files are available from the XXX database (accession number(s) XXX, XXX.).*<br>• If the data are all contained **within the manuscript and/or Supporting Information files**, enter the following: *All relevant data are within the manuscript and its Supporting Information files.*<br>• If neither of these applies but you are able to provide **details of access elsewhere**, with or without limitations, please do so. For example:<br><br>*Data cannot be shared publicly because of [XXX]. Data are available from the XXX Institutional Data Access / Ethics Committee (contact via XXX) for researchers who meet the criteria for access to confidential data.*<br><br>*The data underlying the results presented in the study are available from (include the name of the third party* | All relevant data are within the manuscript and its Supporting Information files. |

| | |
|---|---|
| *and contact information or URL).*<br>• This text is appropriate if the data are owned by a third party and authors do not have permission to share the data.<br><br>* typeset | |
| Additional data availability information: | Tick here if your circumstances are not covered by the questions above and you need the journal’s help to make your data available. |

*Powered by Editorial Manager® and ProduXion Manager® from Aries Systems Corporation*

Manuscript Click here to access/download;Manuscript;Manuscript.docx

# Drivers of Rodent Community Structure in an Urban National Park, Kenya.

Immaculate M. Mungai[1,2¶], Nathan Gichuki[1], Dorcus A.O. Sigana[1], Benard Agwanda[2], Patrick Chiyo[3], Vincent Obanda[4¶], Olivia Wesula Lwande[5¶*]

## Institutional affiliations

[1]Department of Biology, University of Nairobi, Kenya

[2]Department of Mammalogy, National Museums of Kenya, Kenya

[3]Institute of Primate Research, Kenya

[4] Department Veterinary Science and Laboratories, Wildlife Research & Training Institute, Kenya

[5]Department of Clinical Microbiology, Umeå University, Sweden

Emails:

immaculate2030@gmail.com

ngichuki@uonbi.ac.ke

dsigana@uonbi.ac.ke

benrisky@gmail.com

pchiyo@gmail.com

vobanda@gmail.com

olivia.lwande@umu.se

Corresponding author: olivia.lwande*

**Key words**: rodent diversity, rodent abundance, habitat structure, population composition, ecosystem, Urbanization

# Abstract

Nairobi National Park's ecosystem is among Kenya's most vulnerable because of the extreme pressure of urbanization. Understanding the structure of the rodent community in Nairobi National Park is important because rodents are sensitive bioindicators of ecosystem health. The association of vegetation type, vegetation metrics, seasonality, and anthropogenic disturbance with rodent community structure was also evaluated. To achieve this, rodent

samples were collected from 15 sites during the wet and dry seasons to assess rodent diversity and relative abundance. Capture-release method was used to trap rodents in the savannah, forest, and riverine vegetation types of Nairobi National Park during the dry and wet seasons. From 56 rodents trapped, five species identified were *Lemniscomys striatus, Hylomyscus sp, Rattus rattus, Mus mus* and *Otomys tropicalis.* Rodent diversity at NNP was low (Simpson=0.7130; Shannon Weiner=1.40; Brillouin index=1.27) while Pielou's species evenness, was moderate=0.44 indicating near equity in species distribution. Univariate Generalised linear models showed that rodent abundance was influenced by season, vegetation type, and vegetation metrics but not by human disturbance/edge effects. The multivariate model indicated that rodents were abundant in the wet season compared to the dry season, and that abundance was also positively associated with increased tree and shrub densities. Rodent species richness was positively associated with higher tree density, while vegetation types influenced rodent species diversity. Rodent abundance was influenced by vegetation type, vegetation metrics (density and cover), and season but not by human disturbance. It was observed that the diverse anthropogenic activities occurring in NNP, do not significantly influence rodent abundance as compared to the biotic and abiotic factors. Factors that influence vegetation and season, like climate change, are thus a threat to the faunal diversity in NNP. This first rodent survey in this Park, provide preliminary data for continued monitoring of this ecosystem.

# Introduction

Urban protected areas provide vital ecosystem services for the urban population and are natural refuges for biodiversity. However, it appears that urbanization poses a serious risk to the preservation of biodiversity, particularly because it disrupts the organization and operation of ecosystems and communities. Some mammalian species, like rodents, are more sensitive to ecosystem changes caused by humans or the environment, making them suitable bioindicators for assessing the health of ecosystems [1, 2]. Surveys of rodent communities in protected areas in Kenya are common [3-5], except for NNP where such data is scarce. The available information on rodents in Nairobi was obtained about 56 years ago. The survey was conducted before Nairobi City’s extensive expansion, and out of 4,320 trapnights, only *Crocidura fumosa* and *Mastomys coucha* were trapped [6]. There has been no published survey since then. However, NNP is currently ecologically fragile due to being within one of Africa's fastest-growing cities [7] and requires frequent ecosystem health monitoring.

The type and structure of vegetation are essential drivers of rodent community structures, impacting their distribution, species diversity, and abundance [8-10]. Locations with higher herbaceous cover benefit rodents by providing predator protection, food, and optimal microclimatic conditions [11]. Landscape use strategies within and without protected areas appear to generate rapid changes in habitats and directly impact small mammals' community structures [12]. This is because human activities modify the complexity and heterogeneities of landscapes, which are the necessary characteristics that support animal populations, ecological interactions, species diversity, and ecosystem functioning [13-15].

The NNP has experienced increased habitat disturbances in recent years, including the construction of roads, recreational facilities, and substantial housing projects along its borders. Such disturbances typically result in a cascade of negative consequences, including the loss of ecological balance and a consequent decline in the diversity of flora and fauna [16]. Habitat disturbance may result in a considerable loss in shrub cover, which lowers mammalian species richness while increasing overall abundance [17]. However, the impact of habitat disturbance or creation of edge effect is very dynamic and fluctuates across time and location, while responses by animal communities may differ according to microhabitat, season, and species [18]. The community structure of small mammals, especially rodents, is likely to be influenced by the edge habitat, where habitat 'generalists' may predominate over habitat 'specialists' [19].

The purpose of the study was to determine the species diversity and abundance in the three primary vegetation types within NNP and to evaluate the effects of seasonality, vegetation metrics, and habitat disturbance on the parks' rodent population structure.

## Ethical statement

The study was approved by Kenya Wildlife Service, permit number KWS/BRP/5001. The study was non-destructive because used non-lethal traps under the capture and release method.

# Materials and methods

## Study area

The study was carried out at Nairobi National Park (NNP), located seven kilometers from the Central Business District of Nairobi City, a protected area in Nairobi County (Fig 1). The park is electric fenced except on its southern part, which is left open as a corridor for animal migration. Recently, the park has been impacted by several heavy infrastructural developments, including the construction of a tarmac road highway along the southern bypass, the Standard Gauge Railway line and human settlements at the park boundary besides occupied areas inside. The annual amount of rainfall varies between years and is bimodal, with heavy rainfall from March to May and relatively lesser amounts of rainfall from October to December. Ogega et al., (2019) reported that between 2000 and 2017, the intra-seasonal rainfall variability increased [20]. According to Ng'ang'a, (1992), the temperature fluctuates between the warm season in December to April, and the cool period extends from June to August. Relative humidity oscillates between 55% during the day and increases to 80% at night [21]. The vegetation is structured, with the largest area characterized by the open grasslands dominated by *Pennisetum mezianum, Setaria phleoides, Themeda triandra, Digitaria macroblephora* grass species, as well as scattered low-canopy *Vachellia drepanolobium* [22] The second largest vegetation type is the open woodland forest, mostly on the elevated areas west of NNP and the riverine woodland along the several streams in the park. Wildlife diversity includes herbivores (antelopes, buffalo, giraffes, wildebeest, zebra, rhinoceros), and carnivores (hyenas, lions, and jackals).

**Figure 1: The Nairobi National Park with the sampling points in the Savanna, Forest and Riverine Ecosystems (E – Edge habitat, E1 Athi Basin, E2 Bangla, E3 Mokoiyet, E4 David Sheldrick, E5 Clubhouse, E6 Asian Settlement, C – Sampling points acting as control for the edge habitats, C1- Lion Dip, C2- Kingfisher, C3- Nangolomon Dam, C4- Nairobi Tented Camp, C5- Nangolomon Circuit, C6- Southern Bypass, EX – extra sampling points within the park, EX1- Nairobi Tented Camp, EX2- Hyena Dam and EX3- Park Point 2B).**

## Rodent community survey

A stratified random design to sample rodents in the three distinct vegetation types in NNP (Savanna open grassland, dry upland forest, and riverine forest or woodland) and a line transect approach as opposed to a grid system. Line transect provides a better resolution of community structure for a given effort [23]. A total of 15 sampling points were distributed across the three main habitats in the park and the sampling points included random areas in each of the main vegetation types, areas close to human settlements and the park boundaries, which represented 'edge habitats'. To determine the effect of 'edge', each transect at the 'edge' had a control point where traps were placed away from 'edge habitats'. 20 Sherman traps were placed at each sampling point in a 100m line of transect. Peanut butter and whole-grain oats were used as baits in the traps, which were deployed for three consecutive nights before relocating to other sites. The Capture-Release method was employed, whereby upon

trap inspection each morning, the trapped rodents were removed from the trap, identification features noted, and marked before release on the same capture site. The identification features of importance were mass, sex, reproductive condition and species' specific traits.

# Tree and Shrub Density and their Percent Cover

## Tree density

Tree density as estimated using the point-centered-quarter (PCQ) method (Mitchell, 2007), whereby trees (plants >5 M in height) within quadrats placed at equidistance along rodent-sampling transects were enumerated and identified to the lowest taxa.

## Shrub density

Shrub density (plants <5 M in height) within quadrats placed at equidistance along rodent-sampling transects were enumerated and identified to the lowest taxa.

## Ground cover

A 30 x 30cm wire quadrat, thrown along each transect, and a visual assessment of the percentage ground cover of the quadrat made. An average for the ground cover along each transect was determined within the three vegetation types in NNP (Savannah, Riverine, and Forest).

## Herb layer

Herb layer was identified and estimated the percent area within 1 x 1 m plots along the transects in each vegetation type (savannah, riverine and forest).

# Data Analysis

## Rodent Species Diversity, Richness, Evenness and Abundance

Rodent diversity indices, Simpson diversity index, Shannon Weiner diversity index, and the Brillouin diversity index were determined for the entire area sampled (all sampling sites combined), for separate sampling sites, vegetation type (forest, savannah, and riverine vegetation), human disturbance (Edges or human-disturbed sites, the adjacent controls, and the pristine Park interior).

The Shannon-Wiener Index (H') is the most used diversity index in ecology, which quantifies the uncertainty associated with predicting a new taxon given the number of taxa and evenness in the abundance of individuals within taxa. It is defined using the following equation:

$$H' = \sum \left( \frac{ni}{N} \times ln \frac{ni}{N} \right)$$

Where - ni is the number of individuals of each of the i species, and N is the total number of individuals at each site.

Values of H′ can range from 0 to 5, although they typically range from 1.5 to 3.5.

The Shannon-Wiener Index assumes that the sample for site was collected randomly.

The Brillouin Index (HB) is a modification of the Shannon-Wiener Index that is preferred when samples are likely not to have been sampled randomly:

$$H_B = \frac{lnN! - \sum \ln ni!}{N}$$

The Simpson's Index (λ), which is the probability that two individuals drawn at random from an infinitely large community will be different species, is a measure of dominance and as such weighs towards the abundance of the most common taxa. Simpson's Index is expressed as the reciprocal (DS=1−λ), hence a measure of diversity, where higher values represent higher diversity. It is less sensitive to rare species than the Shannon-Wiener Index. Simpson's index ranges from 0 to 1, and is defined by the following equations:

$$\lambda = \sum \frac{ni(ni-1)}{N(N-1)}$$

$$D^s = 1 - \sum \frac{ni(ni-1)}{N(N(-1)}$$

These indices were calculated using the "vegan" package of the R statistical software (Oksanen et al 2020). In addition, the differences in diversity across vegetation types, disturbance types and seasons were tested using the Hutcheson student t-tests from the "ecolTest" package [24] in the R software for statistical computing.

## Tree and Shrub Density and their Percent Cover

Each location's density of trees and shrubs, the percent herbaceous cover, and the dominant plant species (≥ 80% cover) were determined. Tree and shrub densities, herbaceous cover and the presence of dominant plant species were first used to characterize their relationships with vegetation type and human habitat disturbance. Second, variation in tree and shrub densities and percent herbaceous cover were used to test their direct influence on rodent diversity.

To characterize habitat variation based on tree and shrub density, herbaceous cover, and dominant species in relation to vegetation type and human disturbance, Principal Components Analysis (PCA) was used to classify vegetation type or habitat disturbance by plotting scores in a two-dimensional biplot. PCA is a scaling procedure that reduces dimensions of complex multivariate data, i.e., shrub and tree densities; herbaceous cover; dominant plant species and projects. These reduced dimensions to a grouping variable to discern habitat classes and their important characteristics.

Additionally, the differences in mean tree and shrub density between vegetation type and human disturbance were tested using Analyses of Variance (one-way ANOVA). However, the differences in herbaceous cover among vegetation types, disturbed and control sites were tested using Kruskal-Wallis tests. The "vegan" package was used in PCA while the core R software [25] was used for ANOVA and Kruskal-Wallis tests.

To test the influence of habitat metrics such as tree and shrub density and herbaceous cover, vegetation type and habitat disturbance on rodent species richness, abundance and diversity, a Generalized Linear Model framework with a Poisson family and a log-link function was used. The rodent species richness, abundance, and diversity (Shannon Weiner diversity index) per site, were incorporated as dependent covariates in three independent models.

The following independent covariates (predictor variables) were used; vegetation metrics (tree density, shrub density, herbaceous vegetation cover), vegetation type (forest, savannah, and riverine vegetation), disturbance (disturbed, control and park), and season (wet and dry periods). Numeric variables, particularly vegetation metrics, were standardized to stabilize the variance of coefficients and to provide unbiased hypothesis testing.

Univariate analysis followed by multivariate analyses and model selection was performed. Model selection was attained by computing the coefficients of all possible simple and complex model combinations using the ‘MuMIn’ package in the R statistical software (Barton 2020). The best model was selected based on Akaike Information Criteria (AIC); the most parsimonious or best model being one with a small AIC.

# Results

## Rodent Species Diversity (Richness, Evenness) and Abundance

Using 20 traps per location for three nights each month over a three-month period, the capture entailed a total of 2700 trap nights. Of the 2700, 65 trap nights were non-effective,

self-triggered or trapped non-target species, leaving 2635 effective trap nights. A total of 56 individual rodents were captured consisting of five species dominated by *Lemniscomys sp* (43%), followed by *Rattus rattus* (23%) and *Otomys tropicalis* (20%) while *Mus mus* (5%) and *Hylomiscus sp* (9%) were less common. The overall rodent diversity in Nairobi NP was low (Simpson = 0.7130102; Shannon Weiner = 1.40, Brillouin index = 1.27). Pielou's species evenness, J was moderate (0.44), indicating nearly average equity in species distribution (Table 1).

**Table 1: Variation in rodent diversity indices: species richness, evenness and rodent abundance by sampling site or location**

| Sampling Location | Simpson ($D^S$) | Shannon-Weiner Index (H') | Brillouin Index ($H_B$) | Species Richness | Pielou's Evenness (J) | Species Abundance |
|---|---|---|---|---|---|---|
| **Asian Settlement** | - | - | - | - | - | 0 |
| **Athi Basin** | 0 | 0 | 0 | 1 | - | 1 |
| **Bangla** | 0 | 0 | 0 | 1 | - | 4 |
| **Club House** | 0.560 | 0.950 | 0.599 | 3 | 0.510 | 5 |
| **David Sheldrick** | 0.305 | 0.483 | 0.395 | 2 | 0.440 | 16 |
| **Ex1** | 0 | 0 | 0 | 1 | - | 3 |
| **Ex2** | 0 | 0 | 0 | 1 | - | 5 |
| **Hyena Dam** | 0 | 0 | 0 | 1 | - | 1 |

| | | | | | | |
|---|---|---|---|---|---|---|
| **Kingfisher** | 0 | 0 | 0 | 1 | - | 6 |
| **Lion Dip** | - | - | - | - | - | 0 |
| **Mokoiyet** | 0 | 0 | 0 | 1 | - | 3 |
| **Nairobi Tented Camp** | 0.568 | 0.937 | 0.691 | 3 | 0.517 | 9 |
| **Nangolomon Circuit** | - | - | - | - | - | 0 |
| **Nangolomon dam** | 0 | 0 | 0 | 1 | - | 3 |
| **Southern Bypass** | - | - | - | - | - | 0 |
| **Grand Total** | **0.713** | **1.394** | **1.267** | **5** | **0.443** | **56** |

Despite identical trapping efforts, rodent abundance and species richness varied across 15 sampling sites investigated in this study. No capture of rodents were recorded from Southern Bypass, Nangolomon Circuit, Lion dip, and Asian settlement. However, the highest number of rodents was captured in the permanent housing areas (David Sheldrick and the Nairobi-tented camp site). Higher species richness per site of rodents were caught at permanent housing areas (Club House, David Sheldrick, and Nairobi Tented Camp sites) compared natural habitats (Ex1, Ex2, Hyena dam, Kingfisher, Athi basin, Bangla, and Nangolomon dam) with only a single species (name). The site with the highest rodent diversity was Nairobi Tented Camp (DS = 0.57, H'= 0.94 and HB = 0.69 followed by Club House (DS = 0.56, H' = 0.95 and HB = 0.56). The David Sheldrick

site had a low species diversity (DS = 0.30, H'= 0.48 and HB = 0.40). The rest of the park had zero diversity.

Forests had a higher species diversity, richness and abundance than either savannah or riverine vegetation, however, species evenness was similar between forest and savannah vegetation (Table 2). There was a statistically significant difference in Shannon Weiner diversity between forest vegetation and savannah (forest = 1.50, savannah = 0.54; Hutcheson t-statistic = 5.98, df = 21.27, P <0.0001) or riverine vegetation (forest = 1.50, riverine = 0.377; Hutcheson t-statistic = 4.68, df = 9.91, P = 0.0009). There was, however, no difference in diversity between savannah and riverine vegetation (savannah = 0.540 and riverine = 0.377; Hutcheson t-statistic = 0.611, df = 14.03, P = 0.551).

**Table 2: Variation in rodent diversity indices, species richness, evenness and rodent abundance across vegetation types and disturbance categories**

| | **Simpson ($D^S$)** | **Shannon-Weiner Index (H')** | **Brillouin Index ($H_B$)** | **Species Richness** | **Pielou's Evenness (J)** | **Species Abundance** |
|---|---|---|---|---|---|---|

| | | | | | | |
|---|---|---|---|---|---|---|
| **Vegetation** | | | | | | |
| **Forest** | 0.754 | 1.500 | 1.313 | 5 | 0.469 | 35 |
| **Riverine** | 0.219 | 0.377 | 0.260 | 2 | 0.316 | 8 |
| **Savanah** | 0.355 | 0.540 | 0.435 | 2 | 0.512 | 13 |
| | | | | | | |
| **Disturbance** | | | | | | |
| **Control** | 0.612 | 1.022 | 0.881 | 3 | 0.558 | 23 |
| **Edge** | 0.640 | 1.146 | 0.990 | 4 | 0.461 | 29 |
| **Park** | 0.375 | 0.562 | 0.347 | 2 | 0.541 | 4 |

Human disturbed sites or edges had a slightly higher Shannon Weiner rodent diversity index than the control sites (Table 2), but the difference was not statistically significant (edge = 1.145, control = 1.022; Hutcheson t-statistic = 0.91, df = 50.38, P = 0.370)

## Habitat variability

The 15 sites sampled were classified into three vegetation types namely, Savannah (Asian Settlement, Bangla, Ex1, Kingfisher and Southern Bypass), Forest (Club House, David Sheldrick, Ex2, Nairobi Tented Camp, and Nangolomon Circuit), and Riverine (Athi Basin, Hyena

Dam, Lion Dip, Mokoiyet, and Nangolomon Dam). These sites were further classified as human-disturbed areas, Control (undisturbed areas in the park with similar vegetation characteristics and at an average distance of 3 km from the disturbed site), and the Park (general undisturbed areas). The edge or disturbed sites consisted of (Asian Settlement, Athi Basin, Bangla, Club House, David Sheldrick, Mokoiyet, and Southern road bypass), while the control sites consisted of (Kingfisher, Lion Dip, Nairobi Tented Camp, Nangolomon Circuit, and Nangolomon Dam). In the areas classified Park (undisturbed), additional site named Ex1, Ex2 and Hyena Dam was sampled for each vegetation type; savanna, forest and riverine.

Principal Component Analysis of trees & shrub densities and herbaceous cover from 15 sites belonging to the three vegetation classes resulted in three principal axes accounting for 100% of the variance, with the first two axes responsible for about 93% of the variance. On the other hand, PCA results based on the dominant plant species in each sampling site produced 15 principal axes from the presence and absence of 31 plant species. Twelve of these principal components explained 100% of the variance, with 6 principal components explaining 90% of the variance. Projecting vegetation types and habitat disturbance groupings on PCA axes revealed that vegetation types could be discriminated from habitat metrics (Fig 2A and 2B) but not human disturbance (Fig 2C and 2D).

**Fig 2: Principal component analyses biplots showing clustering of vegetation type (A, B) and human disturbance (C, D) using shrub density, tree density, and herbaceous cover (A, C) and species composition (B, D)**

Analyses of Variance revealed that tree and shrub densities varied across vegetation types and habitat disturbance. However, the variation was statistically significant for vegetation types but not between disturbed areas and controls (Table 3, Fig 3). Similarly, Kruskall-Wallis analyses also revealed that median herbaceous cover variations were statistically significant across vegetation types but not across human disturbance categories (Table 3).

**Table 3: The mean and median variation in shrub and tree density per hectare and percent herbaceous cover across vegetation types and human disturbance categories**

| **Habitat classification** | **Vegetation metric** | **Density Mean ± SD** | **Density Median ± MAD** | **Statistics** | **P value** |
|---|---|---|---|---|---|
| **Vegetation** | | | | | |
| **Forest** | Herbaceous cover | 29.00 ± 34.35 | 15 ± 7.41 | | |
| **Riverine** | Herbaceous cover | 93.2 ± 4.15 | 94 ± 5.93 | | |

| | | | | | |
|---|---|---|---|---|---|
| **Savannah** | Herbaceous cover | 68.8 ± 28.63 | 89 ± 1.48 | 7.757 | 0.0207 |
| | | | | | |
| **Forest** | Shrub density | 280 ± 268.33 | 400 ± 296.52 | | |
| **Riverine** | Shrub density | 680 ± 414.73 | 600 ± 593.04 | | |
| **Savannah** | Shrub density | 1440 ± 517.69 | 1200 ± 296.52 | 10.17 | 0.0026 |
| | | | | | |
| **Forest** | Tree density | 194 ± 59.1 | 220 ± 44.48 | | |
| **Riverine** | Tree density | 25 ± 35.36 | 0.00 ± 0.00 | | |
| **Savannah** | Tree density | 15 ± 22.36 | 0.00 ± 0.00 | 28.95 | <0.0001 |
| | | | | | |
| **Disturbance** | | | | | |
| **Control** | Herbaceous cover | 57.17 ± 40.3 | 65 ± 43 | | |

| | | | | | |
|---|---|---|---|---|---|
| **Edge** | Herbaceous cover | 62.14 ± 38.61 | 90 ± 8.9 | | |
| **Park** | Herbaceous cover | 88.5 ± 0.71 | 88.5 ± 0.74 | 0.0902 | 0.9556 |
| | | | | | |
| **Control** | Shrub density | 700 ± 723.88 | 500 ± 593.04 | | |
| **Edge** | Shrub density | 771.43 ± 647.34 | 600 ± 593.04 | | |
| **Park** | Shrub density | 1200 ± 0.00 | 1200 ± 0.00 | 1.124 | 0.357 |
| | | | | | |
| **Control** | Tree density | 111.67 ± 116.05 | 100 ± 148.26 | | |
| **Edge** | Tree density | 71.43 ± 76.96 | 50 ± 74.13 | | |
| **Park** | Tree density | 0.00 ± 0.00 | 0.00 ± 0.00 | 0.451 | 0.648 |

Herbaceous cover, shrub density and tree density were highly variable in controls compared to disturbed sites, but the differences were not statistically significant (Fig 3A - 3C). The herbaceous cover was higher in riverine and savannah vegetation compared to forests (Fig 3D). It was also noted that shrub density was highest in savannah and lowest in forest vegetation, but it was intermediate in riverine habitats (Fig 3E). In contrast, tree density was highest in forest vegetation but was lower in savannah and riverine vegetation (Fig 6F).

**Fig 3. Boxplots showing the variation in herbaceous cover, shrub density, tree density with respect to habitat disturbance (A, B, C) and Vegetation type (D, E, F) in NNP**

Dominant plant species composition varied across vegetation types (Table 4). The grass species (*Themeda trianda)* was the primary vegetation in all the sites in Savannah. However, the dominant herb species in the savannah included *Lippia* sp., *Ocimum suave* and *Solanum incanum,* whereas the dominant trees were *Searsia natalensis*, and *Vachellia drepanolobium*.

**Table 4. The percent distribution of dominant plant species in sites sampled by vegetation type and by disturbance type in NNP.**

| **Plant classification** | | | **Vegetation** | | | **Disturbance** | | |
|---|---|---|---|---|---|---|---|---|
| **Species** | **Abbre1** | **Growth form** | **Forest** | **Riverine** | **Savannah** | **Control** | **Edge** | **Park** |

| | | | | | | | | |
|---|---|---|---|---|---|---|---|---|
| *Themeda triandra* | Thtr | G | 20 | 80 | 100 | 67 | 57 | 100 |
| *Cyperus rotundus* | Cyro | G | 40 | 20 | 20 | 67 | 0 | 0 |
| *Cynodon dactylon* | Cyda | G | 20 | 20 | 40 | 17 | 43 | 0 |
| *Panicum sp* | Pasp | G | 40 | 0 | 0 | 33 | 0 | 0 |
| *Chloris gayana* | Chga | G | 20 | 0 | 0 | 17 | 0 | 0 |
| *Eragrostis sp* | Ersp | G | 20 | 0 | 0 | 17 | 0 | 0 |
| *Lippia sp* | Lisp | H | 0 | 40 | 100 | 17 | 57 | 100 |
| *Ocimum suave* | Ocsu | H | 0 | 40 | 100 | 33 | 43 | 100 |
| *Solanum incanum* | Solin | H | 0 | 40 | 100 | 33 | 43 | 100 |
| *Hibiscus sp* | Hisp | H | 0 | 40 | 40 | 0 | 29 | 100 |
| *Justicia sp* | Jusp | H | 0 | 20 | 40 | 33 | 14 | 0 |
| *Aspilia mossambicensis* | Asmo | H | 0 | 0 | 40 | 17 | 14 | 0 |
| *Nymphaea nouchali* | Nyno | H | 20 | 0 | 20 | 33 | 0 | 0 |
| *Asparagus sp* | Assp | H | 0 | 0 | 40 | 17 | 14 | 0 |
| *Parthenium hysterophorus* | Pahy | H | 0 | 0 | 40 | 17 | 14 | 0 |
| *Hypenia sp* | Hypa | H | 40 | 0 | 0 | 33 | 0 | 0 |
| *Hyphaene sp* | Hype | S | 0 | 0 | 20 | 0 | 14 | 0 |
| *Phyllanthus nummulariifolius* | Phnu | S | 40 | 20 | 40 | 67 | 14 | 0 |

| | | | | | | | | |
|---|---|---|---|---|---|---|---|---|
| *Lantana camara* | Laca | S | 40 | 0 | 40 | 33 | 29 | 0 |
| *Opuntia sp* | Opsp | S | 0 | 0 | 40 | 17 | 14 | 0 |
| *Dovyalis caffra* | Dvca | S | 20 | 0 | 20 | 0 | 29 | 0 |
| *Carissa spinarum* | Casp | S | 20 | 0 | 0 | 17 | 0 | 0 |
| *Searsia natalensis* | Sena | T | 20 | 60 | 100 | 50 | 57 | 100 |
| *Vachellia drepanolobium* | Vadr | T | 0 | 80 | 80 | 50 | 43 | 100 |
| *Croton megalocarpus* | Crme. | T | 80 | 0 | 0 | 33 | 29 | 0 |
| *Olea Africana* | Olaf | T | 80 | 0 | 0 | 50 | 14 | 0 |
| *Croton macrostachyus* | Crma | T | 80 | 0 | 0 | 50 | 14 | 0 |
| *Vachellia gerrardii* | Vage | T | 0 | 0 | 40 | 17 | 14 | 0 |
| *Balanites aegyptiaca* | Baae | T | 0 | 0 | 40 | 17 | 14 | 0 |
| *Senegalia mellifera* | Seme | T | 0 | 20 | 0 | 0 | 14 | 0 |

Key: [1]Abbrev - abbreviations of species names as used in the PCA biplot: G – ground cover; H –herb; S – shrub ; T – tree;

In contrast to Savannah, the forest vegetation was dominated by different tree species, including *Croton megalocarpus*, *C. macrostachys* and *Olea europaea subsp cuspidate*.

The species composition for the riverine vegetation somewhat overlapped with savannah vegetation. The primary grass species in the riverine was also *Themeda trianda*, whereas the

dominant trees were *Searsia natalensis* and *Vachellia drepanolobium* (Table 4). It was noted that some plants were restricted to particular vegetation types. The grasses restricted to the forest included *Panicum* sp., *Chloris gayana*, and *Eragrostis sp*. while the trees restricted to the forest included *Croton megalocarpus*, *Olea europaea subsp cuspidate* and *Croton macrostachyus*. Species restricted to savannah were *Vachellia gerrardii* and *Balanites aegyptiaca* among trees, *Opuntia* sp., and *Hyphaene* sp., among shrubs and *Asparagus* sp., *Parthenium hysterophorus* and *Aspilia mossambicensis* among herbs. No grass species was restricted into a savannah vegetation. *Senegalia mellifera* was the only tree restricted to riverine vegetation.

There was limited conspicuous differentiation by plant species composition across habitat disturbance categories. The dominant species in the control sample sites for human disturbance included *Themeda trianda* and *Cyperus rotundus* among grasses, *Phyllanthus nummulariifolius* var. *capillaris* among shrubs and *Searsia natalensis*, *Vachellia drepanolobium*, *Olea europaea subsp. cuspidate* and *Croton macrostachyus* among the trees.

Human-disturbed habitats (edges) also had *Themeda trianda* as a dominant grass, *Lippia* spp as a dominant herb and *Searsia natalensis* as dominant tree. The plants dominating the park were grass species *Themeda trianda* , herb species *Lippia* sp., *Ocimum suave*, *Solanum incanum* and *Hibiscus* sp., while *Searsia natalensis* and *Vachellia drepanolobium* were among the dominant tree species in the park as a pristine category (Table 4).

It was observed that 'control sites' for the 'edge habitats' had more restricted species such as *Cyperus rotundus*, *Chloris gayana*, *Eragrostis sp* among grasses, *Nymphaea nouchali*,

*Hypenia* sp among the herbs and *Carissa spinarum* among the shrubs. *Hyphaene* sp., *Dovyalis caffra* and *Senegalia mellifera* were the plant species restricted to the edge or disturbed habitats (Table 4).

## The influence of vegetation metrics, vegetation type, human disturbance, and seasonality on rodent abundance, species richness, and diversity.

Univariate Generalized Linear Models revealed that the abundance of rodents in this study was influenced by season, vegetation type, and vegetation metrics (density and cover) but not human disturbance (Table 5). The best multivariate model, however, indicated that rodents were abundant in the wet season compared to the dry season, and abundance was also positively associated with increased tree and shrub densities (Table 5, S1 Fig).

**Table 5: Univariate models and the best multivariate model explaining rodent abundance in Nairobi National Park, Kenya**

| | ***Estimate*** | ***Std. Error*** | ***z value*** | ***Pr(>\|z\|)*** | ***AIC*** |
|---|---|---|---|---|---|
| ***Univariate models*** | | | | | |
| ***Intercept*** | 0.385 | 0.163 | 2.356 | 0.0185 | |

| | | | | | |
|---|---|---|---|---|---|
| ***Tree density*** | 0.667 | 0.12<br>8 | 5.20<br>8 | 0.0000 | 119.<br>0 |
| | | | | | |
| ***Intercept*** | 1.253 | 0.16<br>9 | 7.41<br>1 | 0.0000 | |
| ***Riverine cf. forest*** | -1.476 | 0.39<br>2 | -<br>3.76<br>6 | 0.0002 | |
| ***Savannah cf. forest*** | -0.990 | 0.32<br>5 | -<br>3.04<br>9 | 0.0023 | 127.<br>8 |
| | | | | | |
| ***Intercept*** | -0.143 | 0.27<br>7 | -<br>0.51<br>6 | 0.6059 | |
| ***Wet season cf. Dry season*** | 1.196 | 0.31<br>7 | 3.77<br>9 | 0.0002 | 129.<br>9 |
| | | | | | |
| ***Intercept*** | 0.480 | 0.15<br>3 | 3.14<br>4 | 0.0017 | |

| | | | | | |
|---|---|---|---|---|---|
| ***Herbaceous cover*** | -0.537 | 0.138 | -3.899 | 0.0001 | 130.9 |
| | | | | | |
| ***Intercept*** | 0.577 | 0.140 | 4.11 | 0.0000 | |
| ***Shrub density*** | -0.325 | 0.154 | -2.109 | 0.0350 | 142.0 |
| | | | | | |
| ***Intercept*** | 0.651 | 0.209 | 3.12 | 0.0018 | |
| ***Edge cf. Control*** | 0.078 | 0.279 | 0.278 | 0.7809 | |
| ***Park cf. Control*** | -0.651 | 0.542 | -1.201 | 0.2298 | 146.6 |
| | | | | | |
| ***Best model*** | | | | | |
| ***Intercept*** | -0.403 | 0.296 | -1.361 | 0.1736 | |

| | | | | | |
|---|---|---|---|---|---|
| ***Wet season cf. Dry season*** | 1.168 | 0.317 | 3.69 | 0.0002 | |
| ***Tree density*** | 0.873 | 0.184 | 4.732 | 0.0000 | |
| ***Shrub density*** | 0.326 | 0.190 | 1.715 | 0.0864 | 104.0 |

Rodent species richness was only positively associated with higher tree density (Table 6). Multivariate model selection indicated that there was no better model than a univariate tree density model (Table 6, S2 Fig). The model, including vegetation type and intercept, was also a supported model explaining rodent species richness in NNP (S2 Fig).

**Table 6: Univariate models explaining the association between specific variables with rodent species richness in Nairobi National Park, Kenya**

| | ESTIMATE | STD. ERROR | Z VALUE | PR(>\|Z\|) | AIC |
|---|---|---|---|---|---|
| **INTERCEPT** | -0.348 | 0.225 | -1.550 | 0.1210 | |
| **TREE DENSITY** | 0.397 | 0.195 | 2.037 | 0.0417 | 66 |
| | | | | | |
| **INTERCEPT** | -0.331 | 0.222 | -1.493 | 0.1355 | |
| **HERBACEOUS COVER** | -0.362 | 0.208 | -1.740 | 0.0818 | 66.9 |
| | | | | | |
| **INTERCEPT** | 0.182 | 0.289 | 0.632 | 0.5280 | |
| **RIVERINE CF. FOREST** | -0.876 | 0.532 | -1.645 | 0.1000 | |
| **SAVANNAH CF. FOREST** | -0.693 | 0.500 | -1.386 | 0.1660 | 68.4 |
| | | | | | |
| **INTERCEPT** | -0.511 | 0.333 | -1.532 | 0.1250 | |
| **WET SEASON CF. DRY SEASON** | 0.442 | 0.427 | 1.034 | 0.3010 | 68.9 |
| | | | | | |
| **INTERCEPT** | -0.271 | 0.210 | -1.294 | 0.1960 | |
| **SHRUB DENSITY** | -0.110 | 0.221 | -0.498 | 0.6190 | 69.7 |

| | | | | | |
|---|---|---|---|---|---|
| **INTERCEPT** | -0.288 | 0.333 | -0.863 | 0.3880 | |
| **EDGE CF. CONTROL** | 0.047 | 0.450 | 0.104 | 0.9180 | |
| **PARK CF. CONTROL** | 0.000 | 0.667 | 0.000 | 1.0000 | 72 |

Univariate linear regression model selection using AIC revealed that the best model for species diversity estimated using the Shannon-Weiner index (Table 7), Brillouin index (Table 8) or Simpson index (Table 9) was vegetation type. Specifically, forest vegetation had higher species diversity than either savannah or riverine vegetation. On the other hand, the best overall model selected after evaluating all simple and complex multivariable models (S3-S5 Figs) was a univariate model with diversity positively influenced by tree density (Table7 and 8).

The human disturbance had no influence on diversity estimated using the Shannon-Weiner diversity index (Table 6), Brillouin index (Table 7) or Simpson index (Table 8).

**Table 7: Univariate models for Shannon-Weiner Index of rodent species diversity**

| | **ESTIMATE** | **STD. ERROR** | **T VALUE** | **PR(>\|T\|)** | **AIC** |
|---|---|---|---|---|---|
| **INTERCEPT** | 0.592 | 0.138 | 4.292 | 0.0026 | |

| | | | | | |
|---|---|---|---|---|---|
| **RIVERINE CF. FOREST** | -0.592 | 0.195 | -3.035 | 0.0162 | |
| **SAVANNAH CF. FOREST** | -0.592 | 0.211 | -2.810 | 0.0229 | 7.4 |
| | | | | | |
| **INTERCEPT** | 0.215 | 0.099 | 2.182 | 0.0570 | |
| **TREE DENSITY** | 0.232 | 0.104 | 2.241 | 0.0518 | 10.4 |
| | | | | | |
| **INTERCEPT** | 0.215 | 0.110 | 1.954 | 0.0825 | |
| **SHRUB DENSITY** | -0.173 | 0.116 | -1.497 | 0.1686 | 12.9 |
| | | | | | |
| **INTERCEPT** | 0.215 | 0.120 | 1.802 | 0.1050 | |
| **HERBACEOUS COVER** | -0.094 | 0.125 | -0.748 | 0.4740 | 14.7 |
| | | | | | |
| **INTERCEPT** | 0.234 | 0.208 | 1.126 | 0.2930 | |
| **EDGE CF. CONTROL** | 0.052 | 0.279 | 0.188 | 0.8560 | |
| **PARK CF. CONTROL** | -0.234 | 0.360 | -0.650 | 0.5340 | 16.4 |

**Table 8: Univariate models for Brillouin Index for rodent species diversity**

<table>
<tr><th></th><th>ESTIMATE</th><th>STD. ERROR</th><th>T VALUE</th><th>PR(>|T|)</th><th></th></tr>
<tr><td>INTERCEPT</td><td>0.422</td><td>0.094</td><td>4.484</td><td>0.0020</td><td>-1.06</td></tr>
<tr><td>RIVERINE CF. FOREST</td><td>-0.422</td><td>0.133</td><td>-3.171</td><td>0.0132</td><td></td></tr>
<tr><td>SAVANNAH CF. FOREST</td><td>-0.422</td><td>0.144</td><td>-2.936</td><td>0.0188</td><td></td></tr>
<tr><td></td><td></td><td></td><td></td><td></td><td></td></tr>
<tr><td>INTERCEPT</td><td>0.153</td><td>0.066</td><td>2.336</td><td>0.0443</td><td>1.46</td></tr>
<tr><td>TREE DENSITY</td><td>0.176</td><td>0.069</td><td>2.553</td><td>0.0310</td><td></td></tr>
<tr><td></td><td></td><td></td><td></td><td></td><td></td></tr>
<tr><td>INTERCEPT</td><td>0.153</td><td>0.076</td><td>2.009</td><td>0.0754</td><td>4.77</td></tr>
<tr><td>SHRUB DENSITY</td><td>-0.126</td><td>0.080</td><td>-1.576</td><td>0.1494</td><td></td></tr>
<tr><td></td><td></td><td></td><td></td><td></td><td></td></tr>
<tr><td>INTERCEPT</td><td>0.153</td><td>0.084</td><td>1.830</td><td>0.1010</td><td>6.83</td></tr>
<tr><td>HERBACEOUS COVER</td><td>-0.063</td><td>0.088</td><td>-0.722</td><td>0.4890</td><td></td></tr>
<tr><td></td><td></td><td></td><td></td><td></td><td></td></tr>
<tr><td>INTERCEPT</td><td>0.173</td><td>0.145</td><td>1.189</td><td>0.2680</td><td>8.53</td></tr>
<tr><td>EDGE CF. CONTROL</td><td>0.026</td><td>0.195</td><td>0.134</td><td>0.8970</td><td></td></tr>
</table>

| | | | | |
|---|---|---|---|---|
| **PARK CF. CONTROL** | -0.173 | 0.252 | -0.687 | 0.5120 |

**Table 9: Univariate models for the Simpson index of rodent species diversity**

| | Estimate | Std. Error | t value | Pr(>\|t\|) | AIC |
|---|---|---|---|---|---|
| Intercept | 0.358 | 0.082 | 4.361 | 0.0024 | -4.0 |
| Riverine cf. forest | -0.358 | 0.116 | -3.083 | 0.0150 | |
| Savannah cf. forest | -0.358 | 0.125 | -2.855 | 0.0213 | |
| | | | | | |
| Intercept | 0.130 | 0.059 | 2.222 | 0.0534 | -1.0 |
| Tree density | 0.142 | 0.061 | 2.312 | 0.0460 | |
| | | | | | |
| Intercept | 0.130 | 0.066 | 1.973 | 0.0799 | 1.6 |
| Shrub density | -0.105 | 0.069 | -1.522 | 0.1623 | |
| | | | | | |
| Intercept | 0.130 | 0.072 | 1.814 | 0.1030 | 3.4 |
| Herbaceous cover | -0.057 | 0.075 | -0.754 | 0.4700 | |
| | | | | | |
| Intercept | 0.142 | 0.125 | 1.137 | 0.2880 | 5.2 |

| | | | | | |
|---|---|---|---|---|---|
| Edge cf. Control | 0.031 | 0.168 | 0.185 | 0.8580 | |
| Park cf. Control | -0.142 | 0.216 | - 0.657 | 0.5300 | |

# Discussion

## Patterns of rodent diversity (richness, evenness) and abundance by sampling site, vegetation type and human disturbance

The NNP has five rodent species, of which three are native (*L. striatus*, *Otomys tropicalis*, *Hylomyscus* sp) while two are invasive/exotic commensal species (*Mus mus,* and *Rattus* sp*).* The dominance of *L. striatus* (striped grass mouse) in NNP with 43.9% (25/57) suggests its adaptability to the current ecological state of the park. Specifically, it was noted that *L. striatus* occurred across the three primary vegetation types in NNP, while the Pielou's species evenness was moderate (0.44), indicating nearly average equity in species distribution. The *L. striatus* is one of the common rodents in grasslands, savannah, and cultivated areas, with reports showing that it has adapted to disturbed habitats [26, 27]. The degree to which human interference changes habitat structure, the spatial arrangement of physical things in the landscape, impacts several ecological factors [13]. Therefore, the prevalent human activities, including the infrastructure projects such as railway lines and road networks, recreational places, and the rising levels of air, water and noise pollution in and around NNP, account for a

significant ecosystem disturbance. According to Casula et al (2017), habitat disturbance that modifies habitat structure affects small mammal populations [13]. Overall, human disturbance seems to drive local extirpation of rodent species, especially in urbanized habitats [28, 29].

Considering that there is no documentation on the previous diversity of rodent species in the NNP for comparison with our findings, it is sufficient to conclude that *L. striatus, O. tropicalis,* and *Hylomyscus* sp. are the natural remnant species of the NNP. Only *Crocidura fumosa* and *Mastomys coucha* were caught in four, 320-night traps at Embakasi Plains, the only place with a report on rodent species close to NNP [6]. Because the genus *Mastomys* is found throughout Africa, with *M. natalensis* being most common in East and Central Africa, its absence in NNP was unexpected and may imply local population extinction.

The genus *Hylomyscus* (wood mice) are montane specialists, and were not expected in NNP. *H. endorobae* and *H.kerbispeterhansi* occur in tropical African lowland and montane rainforests, including Mt. Kenya and Mt. Elgon [4, 30, 31], while *H. denniae* are endemic to the Mau escarpment in Kenya (Demos et al., 2014). Perhaps the *Hylomyscus* sp. in NNP could be a distinct species, given that recent surveys and genetics have revealed more species within the genus [32].

The rodent species richness in NNP was comparable to the Laikipia rangeland, Kenya, where richness ranged from 5 - 7 [33]. In contrast, species richness is higher in some other African savannah landscapes. In Katavi and Mikumi National Parks, both in Tanzania, five and 21 species were observed [26, 34], while Nechisar National Park, Ethiopia, had 20 species [35]. Higher rodent abundance and diversity were observed in human-settled sites or

disturbed/edge habitats. Since most of the human-occupied sites were in the forested areas of NNP and because forest vegetation types had higher rodent diversity, richness, and abundance than savannah vegetation types, both vegetation type and human disturbance likely affected rodent population metrics. According to Jeffrey (1977), clearing forests and replacing the patches with domestic housing and cultivation increases the diversity and abundance of rodents [36]. Generally, forest vegetation hosts higher rodent diversity than savannah. Olayemi and Akinpelu (2008) observed a similar pattern in Nigeria, where Shannon-Weiner Index was higher in the forest (H = 1.68) compared to the derived Savannah (H = 0.97) [37]. This pattern is consistent with observations at Mikumi National Park (Tanzania), Bwindi Impenetrable National Park and Kibale National Park (Uganda), where the evergreen forest showed the highest species diversity, compared to the Savanna woodlands [34, 38, 39]. It is posited that evergreen forests support a variety of food resources and provide several microhabitats which may offer cover and nest sites to different small mammal species [38, 39].

## Factors driving rodent abundance, species richness and diversity.

Multiple biophysical factors, such as predator risks and avoidance opportunities, intra- and inter-species competitions, resource quantity and quality, and especially the availability of water and food, influence rodent community composition, richness, abundance and diversity [40]. In NNP, rodent abundance was influenced by season, vegetation type and vegetation metrics (density and cover).

The rodents were abundant in the wet season compared to the dry season, which is a pattern consistent with African rodent communities [41]. Availability of rainfall is highly correlated with the reproduction of small mammals in Africa [42, 43], particularly with the breeding and population dynamics of several rodent species [44]. It was also established that abundance was positively associated with density and cover, especially tree and shrub density in NNP. The spatial distribution of rodents in a landscape is determined by intrinsic and ecological factors, as each species responds differently to habitat structural variability [45]. Rainfall and habitat productivity are interdependent and drive rodent abundance and richness in NNP. Rainfall may directly influence vegetation height and density, hence the resulting cover, which are components of suitable habitats for rodents [41, 46]. Less vegetation cover increases predation risk, reduces food quality, and advances negative competition, eventually affecting population performance [47, 48].

Vegetation type significantly influences rodent species diversity, as supported by the Shannon-Weiner index, Brillouin index, or Simpson index. As the tree density increased, the more diverse was the rodent diversity in NNP.  Less species diversity was expected in the cleared patches for human settlements and infrastructure development in the NNP [17], but instead, these disturbances had little effect on rodent diversity in the park. The findings of this study distinguish between the influence of anthropogenic activities and the effect of the habitat type in which such human activities occur.

In conclusion, the vegetation types influenced rodent diversity, while species richness was only positively associated with higher tree density. Season, vegetation type, and vegetation metrics (density and cover) all influenced rodent abundance but not anthropogenic habitat disturbance.

## Author contribution

The study was conceptualized by IMM, NG, VO and OWL. Field work was performed by IMM, BA and VO. Data analysis was performed by IMM and PC. Critical review of the manuscript was performed by DAOS and OWL. All co-authors contributed in the manuscript.


## Project funding

The study was sponsored by the resources of the National Museums of Kenya. The study was supported by the Swedish Research Council, Research network grant with a focus on Swedish research links, Registration number 2021-05307.


## Declaration conflict of interest

The authors declare no conflict of interest

# Acknowledgement

We appreciate the National Museums of Kenya and the Kenya Wildlife Service for all the logistical support. We also appreciate the research assistants and security rangers for their assistance during fieldwork, especially Mr. Daniel Muteti of Kenya Wildlife Service and the sampling team to the Nairobi National Park while Mr. Jackson King'oo and Mr. Elphas Bitok facilitated the fieldwork. Sincerely appreciation for the logistic and ranger security support from Sergents Evans Ochieng, Joseph Thoya, John Nderitu, and Paul Ngechu (KWS) during field visits to the study sites in Nairobi National Park.


# References


1. Canova L, Fasola M. Small rodents along a ground-cover gradient in coastal Kenya. Tropical Zoology. 2000;13(2):219-26.
2. Mortelliti A, Boitani L. Patterns of rodent species diversity and abundance in a Kenyan relict tropical rainforest. Forest Diversity and management. 2006:365-80.
3. Onditi KO, Song W-Y, Li X-Y, Chen Z-Z, Li Q, He S-W, et al. Patterns and predictors of small mammal phylogenetic and functional diversity in contrasting elevational gradients in Kenya. Frontiers in Ecology and Evolution. 2022;9:742524.
4. Musila S, Monadjem A, Webala PW, Patterson BD, Hutterer R, De Jong YA, et al. An annotated checklist of mammals of Kenya. Zoological Research. 2019;40(1):3.
5. Ogada DL, Peterhans JK, Agwanda B. Preliminary report of shrews and rodents in and around Lake Bogoria National Reserve, Kenya. Journal of East African Natural History. 2009;98(1):129-39.
6. Hartman D. A June-July census of small mammals on the Athi Plains, Kenya. Journal of East African Natural History. 1967;1967(114):1-4.
7. Mundia CN, Aniya M. Analysis of land use/cover changes and urban expansion of Nairobi city using remote sensing and GIS. International journal of Remote sensing. 2005;26(13):2831-49.
8. Williams SE, Marsh H, Winter J. Spatial scale, species diversity, and habitat structure: small mammals in Australian tropical rain forest. Ecology. 2002;83(5):1317-29.
9. Layme VMG, Lima AP, Magnusson WE. Effects of fire, food availability and vegetation on the distribution of the rodent Bolomys lasiurus in an Amazonian savanna. Journal of Tropical Ecology. 2004;20(2):183-7.
10. Holland GJ, Bennett AF. Differing responses to landscape change: implications for small mammal assemblages in forest fragments. Biodiversity and Conservation. 2009;18:2997-3016.
11. Hoffmann A, Zeller U. Influence of variations in land use intensity on species diversity and abundance of small mammals in the Nama Karoo, Namibia. Belgian Journal of Zoology. 2005;135(1):91-6.

12. Panzacchi M, Linnell JD, Melis C, Odden M, Odden J, Gorini L, et al. Effect of land-use on small mammal abundance and diversity in a forest–farmland mosaic landscape in south-eastern Norway. Forest Ecology and Management. 2010;259(8):1536-45.
13. Casula P, Luiselli L, Milana G, Amori G. Habitat structure and disturbance affect small mammal populations in Mediterranean forests. Basic and Applied Ecology. 2017;19:76-83.
14. Gamfeldt L, Snäll T, Bagchi R, Jonsson M, Gustafsson L, Kjellander P, et al. Higher levels of multiple ecosystem services are found in forests with more tree species. Nature communications. 2013;4(1):1340.
15. Hooper DU, Chapin III FS, Ewel JJ, Hector A, Inchausti P, Lavorel S, et al. Effects of biodiversity on ecosystem functioning: a consensus of current knowledge. Ecological monographs. 2005;75(1):3-35.
16. Tews J, Brose U, Grimm V, Tielbörger K, Wichmann MC, Schwager M, et al. Animal species diversity driven by habitat heterogeneity/diversity: the importance of keystone structures. Journal of biogeography. 2004;31(1):79-92.
17. Men X, Guo X, Dong W, Ding N, Qian T. Influence of human disturbance to the small mammal communities in the forests. Open Journal of Forestry. 2015;5(01):1.
18. Flaspohler DJ, Temple SA, Rosenfield RN. Species-specific edge effects on nest success and breeding bird density in a forested landscape. Ecological Applications. 2001;11(1):32-46.
19. Laurance WF, Yensen E. Predicting the impacts of edge effects in fragmented habitats. Biological conservation. 1991;55(1):77-92.
20. Ogega OM, Wanjohi HN, Mbugua J. Exploring the future of Nairobi National Park in a changing climate and urban growth. The Geography of climate change adaptation in urban Africa. 2019:249-72.
21. Ng'ang'a J. The climate and meteorology of Nairobi region, Kenya. 1992.
22. Deshmukh I. Primary production of a grassland in Nairobi National Park, Kenya. Journal of applied ecology. 1986:115-23.
23. Pearson DE, Ruggiero LF. Transect versus grid trapping arrangements for sampling small-mammal communities. Wildlife Society Bulletin. 2003:454-9.
24. Salinas H, Ramirez-Delgado D. ecolTest: community ecology tests. R package. 2021.
25. Team RDC. R: A language and environment for statistical computing. (No Title). 2010.
26. Caro T, Dobson A, Marshall AJ, Peres CA. Compromise solutions between conservation and road building in the tropics. Current biology. 2014;24(16):R722-R5.
27. Ssuuna J, Makundi RH, Isabirye M, Sabuni CA, Babyesiza WS, Mulungu LS. Rodent species composition, relative abundance, and habitat association in the Mabira Central Forest Reserve, Uganda. Journal of Vertebrate Biology. 2020;69(2):20021.1.
28. Wan X, Yan C, Wang Z, Zhang Z. Sustained population decline of rodents is linked to accelerated climate warming and human disturbance. BMC Ecology and Evolution. 2022;22(1):102.
29. Granjon L, Cosson J-F, Quesseveur E, Sicard B. Population dynamics of the multimammate rat Mastomys huberti in an annually flooded agricultural region of central Mali. Journal of Mammalogy. 2005;86(5):997-1008.
30. Happold D, Lock JM. The biotic zones of Africa. Mammals of Africa. 2013;1:57-74.
31. Nicolas V, Colyn M. Seasonal variations in population and community structure of small rodents in a tropical forest of Gabon. Canadian Journal of Zoology. 2003;81(6):1034-46.
32. Nicolas V, Fabre P-H, Bryja J, Denys C, Verheyen E, Missoup AD, et al. The phylogeny of the African wood mice (Muridae, Hylomyscus) based on complete mitochondrial genomes and five nuclear genes reveals their evolutionary history and undescribed diversity. Molecular phylogenetics and evolution. 2020;144:106703.
33. Webala PW, Muriuki G, Lala F, Bett A. The small mammal community of Mukogodo Forest, Kenya. African Journal of Ecology. 2006;44(3):363-70.
34. Venance J. Small mammal communities in the Mikumi national park, Tanzania. Hystrix-the Italian Journal of Mammalogy. 2010;20(2).

35. Workeneh S, Bekele A, Balakrishnan M. Species diversity and abundance of small mammals in Nechisar National Park, Ethiopia. African Journal of Ecology. 2012;50(1):102-8.
36. Jeffrey SM. Rodent ecology and land use in western Ghana. Journal of Applied Ecology. 1977:741-55.
37. Olayemi A, Akinpelu A. Diversity and distribution of murid rodent populations between forest and derived savanna sites within south western Nigeria. Biodiversity and Conservation. 2008;17:2411-25.
38. Isabirye-Basuta G, Kasenene JM. Small rodent populations in selectively felled and mature tracts of Kibale Forest, Uganda. Biotropica. 1987:260-6.
39. Kasangaki A, Kityo R, Kerbis J. Diversity of rodents and shrews along an elevational gradient in Bwindi Impenetrable National Park, south-western Uganda. African Journal of Ecology. 2003;41(2):115-23.
40. Willig MR, Kaufman DM, Stevens RD. Latitudinal gradients of biodiversity: pattern, process, scale, and synthesis. Annual review of ecology, evolution, and systematics. 2003;34(1):273-309.
41. Massawe AW, Mulungu LS, Makundi RH, Dlamini N, Eiseb SJ, Kirsten F, et al. Spatial and temporal population dynamics of rodents in three geographically different regions in Africa: implication for ecologically-based rodent management. African Zoology. 2011;46(2):393-405.
42. Happold D. The small rodents of the forest-savanna-farmland association near Ibadan, Nigeria, with observations on reproduction biology. Rev Zool Afr. 1974;88(4):814-36.
43. Bekele A, Leirs H. Population ecology of rodents of maize fields in central Ethiopia. Belgian journal of zoology/Koninklijke Belgische Vereniging voor Dierkunde-Brussel, 1990, currens. 1997;127(1):38-48.
44. Leirs H, Verhagen R, Verheyen W, Mwanjabe P, Mbise T. Forecasting rodent outbreaks in Africa: an ecological basis for Mastomys control in Tanzania. Journal of Applied Ecology. 1996:937-43.
45. Gentili S, Sigura M, Bonesi L. Decreased small mammals species diversity and increased population abundance along a gradient of agricultural intensification. Hystrix. 2014;25(1):39-44.
46. Bakker E, Olff H, Boekhoff M, Gleichman J, Berendse F. Impact of herbivores on nitrogen cycling: contrasting effects of small and large species. Oecologia. 2004;138:91-101.
47. Keesing F. Impacts of ungulates on the demography and diversity of small mammals in central Kenya. Oecologia. 1998;116:381-9.
48. Flowerdew J, Ellwood S. Impacts of woodland deer on small mammal ecology. Forestry. 2001;74(3):277-87.

Figure
Click here to access/download;Figure;Figures_in_tif_Immaculate_Plos_One

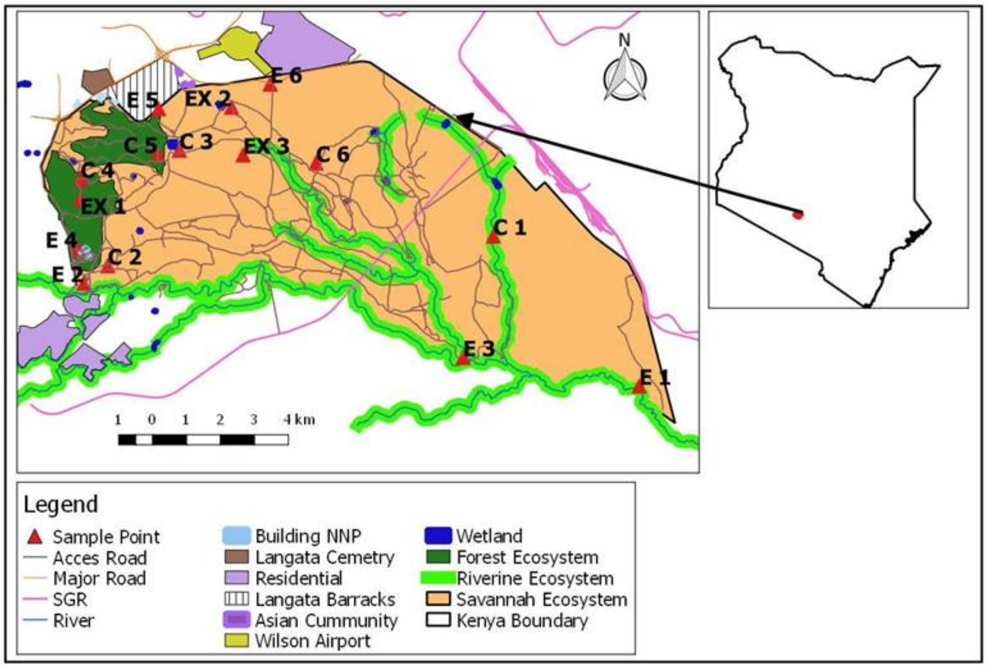


**Figure 1: The Nairobi National Park with the sampling points in the Savanna, Forest and Riverine Ecosystems (E – Edge habitat, E1 Athi Basin, E2 Bangla, E3 Mokoiyet, E4 David Sheldrick, E5 Clubhouse, E6 Asian Settlement, C – Sampling points acting as control for the edge habitats, C1- Lion Dip, C2- Kingfisher, C3- Nangolomon Dam, C4- Nairobi Tented Camp, C5- Nangolomon Circuit, C6- Southern Bypass, EX – extra sampling points within the park, EX1- Nairobi Tented Camp, EX2- Hyena Dam and EX3- Park Point 2B).**

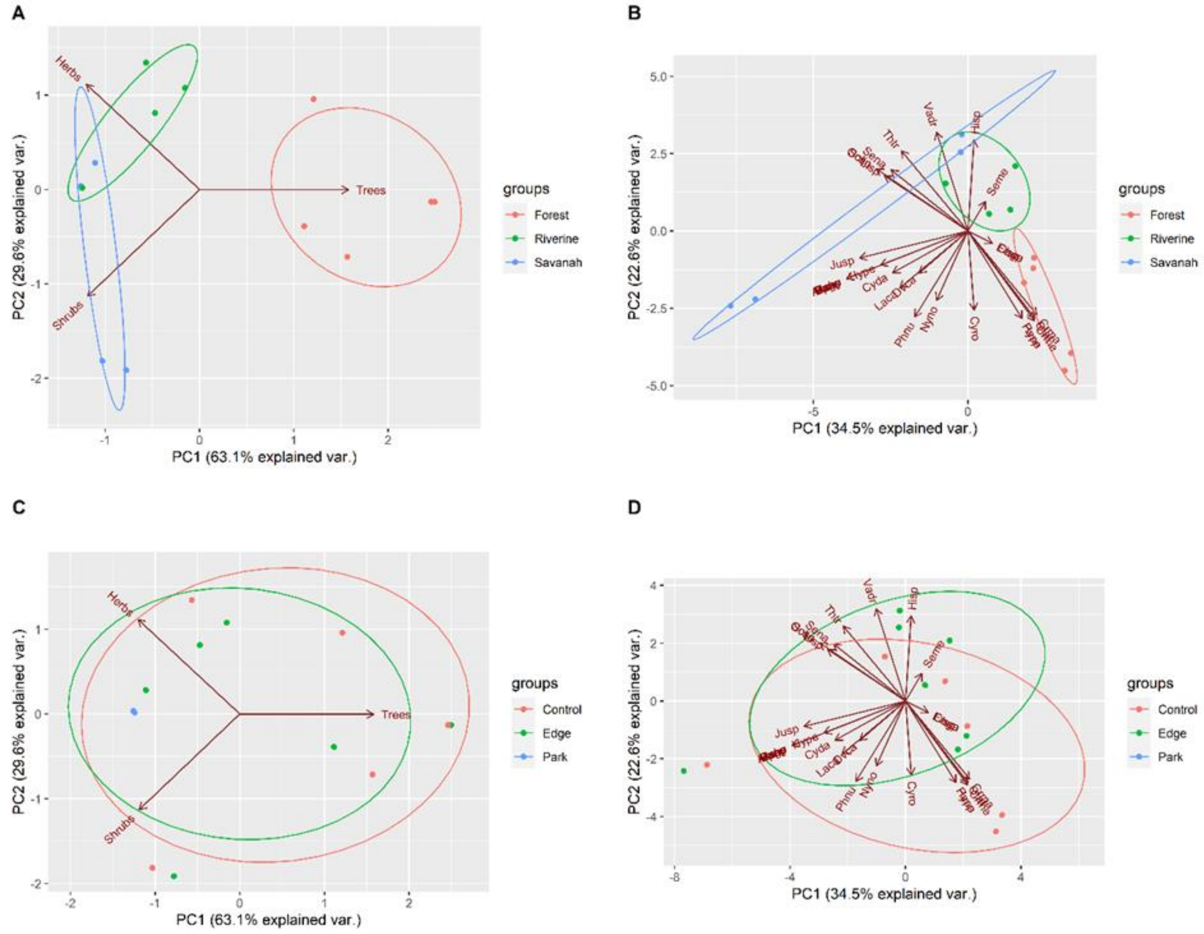


**Figure 2: Principal component analyses biplots showing clustering of vegetation type (A, B) and human disturbance (C, D) using shrub density, tree density, and herbaceous cover (A, C) and species composition (B, D)**

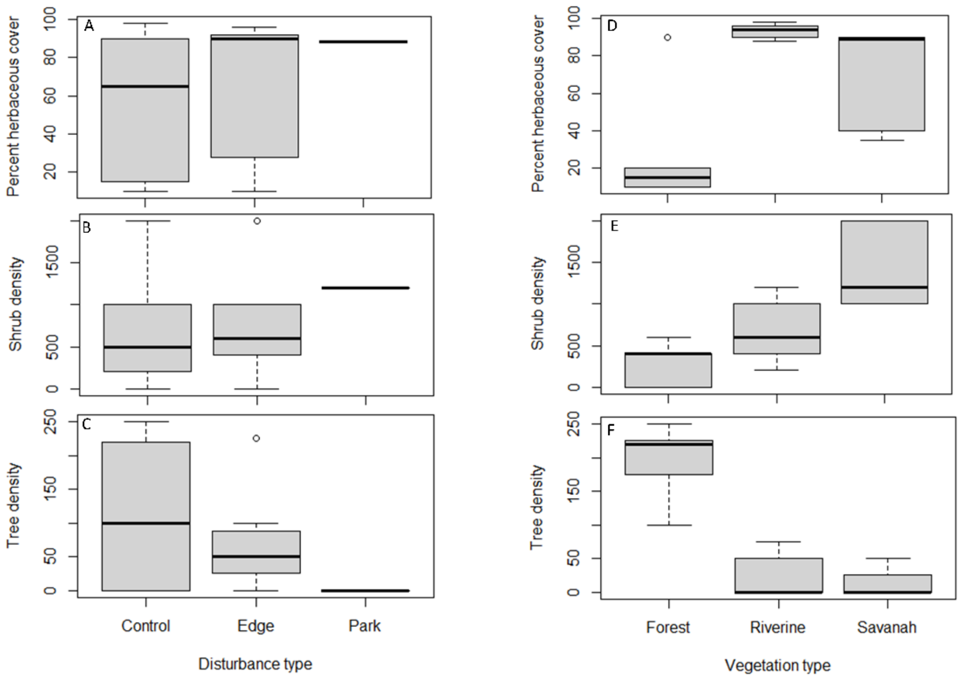


**Figure 3. Boxplots showing the variation in herbaceous cover, shrub density, tree density with respect to habitat disturbance (A, B, C) and Vegetation type (D, E, F) in NNP**

Click here to access/download
**Supporting Information**
Supplimentary_documents_Immaculate_Plos_One.docx
