# Supplementary material for: Drivers of rodent community structure in an Urban National Park, Kenya
Source: PLoS One. 2025 Apr 16;20(4):e0321659. doi: 10.1371/journal.pone.0321659 (PMC12002537; doi:10.1371/journal.pone.0321659)
Supplement: Attachment — Submitted filename: Comments.docx [file pone.0321659.s007.docx]

The current research holds significant importance as it offers insights into a frequently overlooked category of organisms, namely, small mammals. Below are my overall remarks on the manuscript, with more detailed comments provided in the PDF document.

1. **Abstract**: The abstract requires certain adjustments in terms of its writing flow and cohesion. Some of the statements in the abstract appear incomplete. Specifically, it lacks information regarding the statistical methods employed to achieve the research objectives.

2. **Introduction**: The introduction is competently written, but it appears somewhat disjointed and lacks cohesiveness in its narrative. To enhance its clarity, the authors should initially provide a clear definition of the term "Urban protected area." Following this, the first paragraph should incorporate substantial ecological insights into rodents and their pivotal role in maintaining ecosystem health. For instance, rodents serve as crucial prey for meso and small predators, as well as seed predators. The introduction should then delve into how human disturbances and alterations could impact rodent populations and diversity, leading to a discussion of conservation concerns within the National Park. Lastly, it would strengthen the manuscript's scientific rigor to include a hypothesis and corresponding predictions in the conclusion of the introduction.

In the nutshell authors need to make strong ecological rationale with unity and flow in writing.

**3. Methods:**

**Rodent community survey**

The Methods section appears disorganized and lacks a clear direction, leading to confusion for readers. Upon closer examination, it seems that the authors placed transects in various habitats and subsequently employed Sherman trapping. However, the current presentation makes it challenging to comprehend the step-by-step process of their sampling, from habitat selection to rodent capture. To improve clarity, the authors should simplify and provide more detailed explanations of their survey methods, ensuring that the sequence is easy for readers to follow. Additionally, statements like "edge effects" need further elaboration to enhance understanding.

**Tree and Shrub Density and their Percent Cover**

This section should be consolidated under a single heading, such as "Vegetation Variables," as the scattered information under different headings lacks coherence. Additionally, it remains unclear how the quadrates were positioned on the transects—whether they were placed along the transects or centered on Sherman traps. This ambiguity raises concerns regarding the representation of fine-scale rodent habitat usage, which could have substantial implications for the results and their interpretation.

Furthermore, statements like "A 30 x 30cm wire quadrat, thrown along each transect" in line 153 contribute to the confusion in the sampling design.

Moreover, there is insufficient detail provided on how data related to anthropogenic variables was collected during the sampling process. Crucially, the manuscript does not specify how rodent abundance was assessed, whether through traditional capture-recapture methods as briefly mentioned or through alternative methodologies. Clarifications on these points would enhance the comprehensibility of the methodology section and the study as a whole.

Top of Form

**Data analysis**

**Rodent Species Diversity, Richness, Evenness and Abundance**

he rationale behind the inclusion of numerous diversity indices needs clarification in the manuscript. Additionally, there is a lack of information regarding how the richness and evenness indices were calculated. Furthermore, the calculation method for abundance is not clearly stated.

To improve the clarity and conciseness of this section, it would be beneficial for the authors to provide explanations for why they chose to use multiple diversity indices, offer details on how richness and evenness indices were computed, and specify the method employed for calculating abundance. Simplifying the presentation of this information will help readers better understand the study's methodology.

**Tree and Shrub Density and their Percent Cover**

The manuscript would benefit from a clearer rationale for the inclusion of PCA analysis in investigating vegetation differences in various habitats, as it currently appears to create a disconnect between vegetation structure and factors influencing rodent diversity. Staying closely aligned with the study objectives would improve the manuscript's focus. Additionally, it is important for the authors to support their statements with references, especially in the statistical section, to enhance the scientific rigor of their work. The statistical section should not read like a storytelling or blog but should be underpinned by relevant literature and methodology.

Regarding the use of both bivariate and multivariate predictive modeling, the authors should provide a strong justification for this choice. It would be helpful to clarify why they employed both approaches and how they complement each other, especially in cases where GLM with combinations of models may be more powerful. A clear rationale will provide readers with insight into the methodology selection and strengthen the scientific basis of the analysis.

**Results**

The Results section should be rewritten for improved clarity. It's essential to provide a clear explanation of the type or species groupings that the PCA has undertaken.

Furthermore, considering that the main focus of the manuscript is on rodents, it might be best to maintain consistency in the treatment of the vegetation section. Deviating from the core focus on rodents can create distractions, so it's advisable to align the presentation with the central theme of the study.

**Discussion.**

Need more strengthening from the perspective of rodent ecology.

In summary, the manuscript exhibits issues with unity and writing flow, resulting in a fragmented understanding of its sections. There is a perception that the authors have introduced unnecessary objectives related to vegetation and its statistical analysis, which detracts from the central focus on rodents. To enhance the manuscript's potential for publication in PLOS ONE, it is advisable to concentrate solely on the rodent aspect and consider restructuring the manuscript for better scientific coherence. A recent manuscript by Misher et al. (2022) on a similar topic could serve as a helpful reference for organizing the manuscript more effectively. A major revision is recommended to address these concerns and improve the overall quality of the manuscript.

**Misher C, Vats G and Vanak AT (2022) Differential Responses of Small Mammals to Woody Encroachment in a Semi-Arid Grassland. *Front. Ecol. Evol.* 10:755903. doi: 10.3389/fevo.2022.755903**
