# Supplementary material for: Drivers of rodent community structure in an Urban National Park, Kenya
Source: PLoS One. 2025 Apr 16;20(4):e0321659. doi: 10.1371/journal.pone.0321659 (PMC12002537; doi:10.1371/journal.pone.0321659)
Supplement: Attachment — Submitted filename: REBUTTAL 231129.docx [file pone.0321659.s009.docx]

## 29^th^ November 2023

Editor-in-Chief,

Emily Chenette (PhD),

PLOS One.

Dear Emily Chenette,

We are happy to resubmit this research article entitled **“Drivers of Rodent Community Structure in an Urban National Park, Kenya- Manuscript number- PONE-D-23-23260” by** Immaculate M. Mungai, Nathan Gichuki, Dorcus A.O. Sigana, Benard Agwanda, Patrick Chiyo, Vincent Obanda, Olivia Wesula Lwande to **the PLOS One Journal.**

We are grateful for the valuable feedback from the reviewers*.* The comments raised by all reviewers are vital to us - to improve the quality of this manuscript, and for expanding the understanding, not only on how urbanization affects preservation of biodiversity, but also the influence of anthropogenic activities and the effect of the habitat type in which such human activities occur and how these affects the rodent structure.

We hope that our revised manuscript will illuminate some of these important questions better and will be considered for publication in **the PLOS One Journal.**

Please find the point-by-point responses below (in red highlight).

On behalf of all authors,

Olivia Wesula Lwande, corresponding author,
Department of Clinical Microbiology,

901-85 SE, University of Umeå, Sweden
[olivia.lwande@umu.se](mailto:olivia.lwande@umu.se)

**To the Editor, thank you for the extensive and meaningful comments. Find below the reply to the comments raised.**

1. The manuscript has been revised according to PLOS ONE’s formatting style and file names.
2. Language, including grammar and spelling has been checked and revised as appropriate by Dr. George O. Paul ([gomondip@gmail.com](mailto:gomondip@gmail.com) ) with help from the EditScope language check and editing services provider.

- Tracked manuscript included – *supporting Information* file
- Clean copy of the edited manuscript – new *manuscript* file

3. Funding-related text has been deleted from the Manuscript.
4. Ethics statement have been moved to the Methods section.
5. Figure 1 is a map that was created by the author and NOT sourced from a copyright owner.
6. Captions for supporting files have been placed at the end of the manuscript and text citations and file names updated as per the format.

**Reviewer 1**

Line 31: Sentence revised as follows: Nairobi National Park (NNP) is among the most vulnerable ecosystems.

Line 40: The abbreviation NNP is now explained in line 31.

Line 127: The sentence has been revised accordingly.

Line 140: We have given examples of some species-specific traits that may be used in rodent identification in the field. Identification of rodents in the field is based on morphological traits. There are several traits or characteristics that are specific to a species. For instance, some species have hair, while others have fur and others are naked like the naked-mole rats. The hair or fur may be of different colouration/ patterning; The tail may be shorter than the body or longer than the body.

Line 142: We have revised the title as suggested ‘Vegetation Sampling’.

Line 143 -154: Following the change above, and for clarity and readability, this section has been revised.

Line 168 -175: The section has been revised as suggested by the reviewer.

Line 187: Deleted and redrafted in line line 176-177 under Shannon Wieiner index.

Line 206-212: The sentence has been revised.

Line 222-223: Citation has been included.

Line 235-236: Citation has been included.

**Reviewer 2**

*Abstract*:

Line 40-46: describes the statistics used in the study, such as the diversity indices, GLM and both univariate and multivariate models have been mentioned.

*Introduction*: This section has been revised to ensure cohesiveness and flow as guided by the reviewer in their editorial notes and also in the tracked manuscript.

Line 56-57: We have described the Urban Protected area/ National Park and also provided a reference.

Line 57-66: For clarity and flow, we have revised the sentence, combining multiple sentences into one, and providing references.

Line 68-69: Sentence revised.

Line 73-74: Revised by merging with the preceding sentence in line 72-73.

Line 76-77: Sentence revised and reference added.

Line 79-81: Following the major revision, this section is removed.

Line 98: Corrections made as suggested by the reviewer.

Line 101-104: The sentence has been transferred to the Introduction.

Line 104- 106: Rainfall amount has been included in the sentence and also a citation.

Line 114-116: The sentence has been revised – replacing wildlife diversity with ‘Mammals’.

*Methods*

The supplementary data on species abundance are in S1 Table 1 species abundance.

The comments on capture-mark-recapture have been addressed.

Line 127 – 129: The sentence has been revised for clarity.

Line 129-130: We have provided elaboration and included additional citations.

Line 130-134: The sentences have been revised for clarity.

Line 144 -145: Citation format has been corrected according to the journal requirement.

Line 144-145: We have clarified that during sampling, the quadrats were within transects.

Line 153: We have clarified thrown along the transect to ‘thrown over the rodent trapping transects’. Since rodents were captured along specific transects, we wanted to determine the ground cover in the area within the transects.

Line 163-164: We have revised this sentence to show that we used three diversity indices and explained their purpose.

Line 198: The title has been revised – The density of trees and shrubs and herbaceous per cent cover.

Line 206-212: We have included citations.

Line 214-215: We tested differences in densities of trees and shrubs in Savanna, riverine and forest parts of NNP because these parameters may have an association with rodent community (Madden et al, 2019). Our results found that abundance was positively associated with density and herbaceous cover, especially tree and shrub density in NNP.

Line 217: The word ‘core’ has been deleted.

Line 220-223: Sentence revised, and citations included.

Line 241: The sentence has been revised for clarity - ‘self- triggered’ corrected as traps that triggered themselves but failed to capture.

Line 255 -263: These names are sampling locations, which have been provided in Table 1 and their locations are shown in the map (figure 1).

Line 363-373: Transferred to methods and placed under a sub-title – ‘Sampling sites’.

Line 294-313: We have used analysis to demonstrate how the habitat in NNP is variable. The objective of the study is to evaluate the relationship of habitat variability with rodent community structure. We consider this section to be a huge component of the study and if omitted will affect the objective of the study.

Line 315-316: We included Median values, besides the mean, because our large dataset had outliers that might skew the average of the values.

Line 391-393: We have mentioned this analysis in the Data analysis section line 232-233.

Line 424: The sentence has been revised and the word ‘Physical things’ – omitted.

Line 484-490: The discussion on this section have been improved as tracked.
