# Supplementary material for: Drivers of rodent community structure in an Urban National Park, Kenya
Source: PLoS One. 2025 Apr 16;20(4):e0321659. doi: 10.1371/journal.pone.0321659 (PMC12002537; doi:10.1371/journal.pone.0321659)
Supplement: Attachment — Submitted filename: Response to Reviewers.docx [file pone.0321659.s011.docx]

10^th^ August 2024

Dereje Yazezew Mammo, Ph.D.
Academic Editor
PLOS ONE

Dear Dereje Yazezew Mammo,

**RE:** PONE-D-23-23260R1-Drivers of Rodent Community Structure in an Urban National Park, Kenya

Following the comments we reviewed from your esteemed journal dated 16^th^ July 2024. We would like to present our revised manuscript. To our knowledge, we believe we have addressed the additional comments from the academic editor, together with reviewer 1 and 3 as reviewer 2 had already confirmed that we addressed all the comments on the previous draft. We therefore request you to review each comment that we have addressed below in red highlight. I hope you find our article suitable for publication in your Journal.

Sincerely,

Corresponding author,

Olivia Wesula Lwande,

Research Fellow and Docent

Department of Clinical Microbiology,

Umeå University, 901 85 SE, Umeå, Sweden.

Additional Editor Comments:

Thank you for considering PLOS ONE for your manuscript "Drivers of Rodent Community Structure in an Urban National Park, Kenya". Peer review of your manuscript is now complete. Based on the reports and my own reading of your manuscript, I have completed my evaluation of your manuscript.
Although the manuscript is thoughtful in its content and important for conservation endeavor, there are some areas that should be improved prior to publication as indicated by the reviewers. Especially the number of tables should be minimized by substituting with figures as per the feasibility. The references citations and bibliography sections also need revision, for instance the authors used both numbering and author in the citation and the journal names are written inconsistently with a mix of abbreviate and full written. There are also some discursive and self- contradictory run on sentences in the literature and discussion section without logical sequencing, lending to distractions and deviations from the key messages that the authors are trying to convey.

Thank you for your important comments. We reduced the number of tables and corrected the author citations and references. In addition, we clarified numerous comments that appeared to be conflicting and provided suitable abbreviations. Please find a summary of all criticisms addressed, with some particular to reviewer 1 and 3 given that reviewer 2 endorsed the manuscript. We appreciate the time and work put into the review.

Line 32 -35: this section has been rephrased to provide clarity and connectivity to the next paragraphs

Line 37: revised to read – Capture-Mark-Release

Line 38: the words have been deleted

Line 45: the word ‘more’ has been added

Line 52: the words are deleted

Line 110- 112: the sentence has been revised including the citation

Lines 121 – 126: Figure 1: - The map has been revised, removing unnecessary items and rearranging the County and Study area maps. In addition, the title of the figure has provided the necessary description on the maps.

Line 143 – 145: this section describes the methods for trap nights, having been removed from the first sentence of the RESULTS section

Line 202: the version for the R statistical software has been added

Line 229: the capital ‘The’ has been corrected to the small letter ‘the’

Line 251 -252: The sentence has been revised and further information provided as in lines 143 -145 above

Line 262: To reduce the number of tables, Table 1 has been deleted because all the information in this table was already presented in the preceding paragraph.

Lines 260-261: words added to improve sentence clarity

Lines 264 – 265: words added to improve sentence clarity

Line 273: the word has been deleted

Line 286: Table 2 has been converted into 2 graphs and all citations of Table 2 changed to Fig 2 and 3

Line 306: Figure 2 is now named to Figure 4

Line 317: Table 3 is now converted to a graph (Figure 5) to enhance clarity and comparability of data. The statistical significance that was presented in the Table is now cited in the text

Line 329: Figure 3 is now named to Figure 6

Line 337: Table 4 is now converted to a graph (Figure 7) to improve clarity and comparability

Line 378: Table 5 has been revised to remove unnecessary spaces

Line 390: Table 6 has been revised to remove unnecessary spaces

Line 404: Table 7 has been revised to remove unnecessary spaces

Line 409: Table 8 has been revised to remove unnecessary spaces

Line 414: Table 9 has been revised to remove unnecessary spaces

Line 420: The introduction text has been revised to focus on what was possibly detected by this study

Line 432: the words and citations deleted

Line 437-438: Yes, those were the two species present 56 years ago and we did not capture them in this study

Line 474: we inserted the word ‘more’

Line 488: we have replaced ‘or’ with ‘and’

Reviewer #2: This work addresses the often overlooked rodents in comparison to their larger counterparts, highlighting its significance. In the initial manuscript review, I identified several weaknesses and issues, which I outlined. In the current review, the authors have effectively addressed all of my comments, making a compelling case for publication.

Thank you for confirming that we handled all of your comments during the prior evaluation. We are also grateful that you consider our work appropriate for publishing in your respected Journal. Your efforts in reviewing the manuscript are appreciated.

Reviewer #3: Dear authors,
Congratulations on the article. From what I have seen, the manuscript is much improved since the first submission.

Thank you. We appreciate your positive comment.

However, I think the abstract can be improved and I think tables are a bit excessive in the text.
You could transform tables into nice graphs, easier to visualise and compare results (Table 2).
Your figure 1 can hardly be understood by someone who does not know the study area, it should be improved.
In general, all figures and tables could be made more attractive and easier to visualise. The fact that most tables are desformatted makes interpretation difficult.
Overall, these are minor changes that can improve your manuscript and then I believe it is ready for publication.

Thank you for the valuable suggestions and comments. We have now revised the abstract together with the figures and tables as described below:

**Abstract**

Line 32 -35: this section has been rephrased to provide clarity and connectivity to the next paragraphs

Line 37: revised to read – Capture-Mark-Release

Line 38: the words have been deleted

Line 45: the word ‘more’ has been added

Line 52: the words are deleted

Lines 121 – 126: Figure 1: - The map has been revised, removing unnecessary items and rearranging the County and Study area maps. In addition, the title of the figure has provided the necessary description on the maps.

Line 262: To reduce the number of tables, Table 1 has been deleted because all the information in this table was already presented in the preceding paragraph.

Line 286: Table 2 has been converted into 2 graphs and all citations of Table 2 changed to Fig 2 and 3

Line 306: Figure 2 is now named to Figure 4

Line 317: Table 3 is now converted to a graph (Figure 5) to enhance clarity and comparability of data. The statistical significance that was presented in the Table is now cited in the text

Line 329: Figure 3 is now named to Figure 6

Line 337: Table 4 is now converted to a graph (Figure 7) to improve clarity and comparability

Line 378: Table 5 has been revised to remove unnecessary spaces

Line 390: Table 6 has been revised to remove unnecessary spaces

Line 404: Table 7 has been revised to remove unnecessary spaces

Line 409: Table 8 has been revised to remove unnecessary spaces

Line 414: Table 9 has been revised to remove unnecessary spaces
