# Supplementary material for: Drivers of rodent community structure in an Urban National Park, Kenya
Source: PLoS One. 2025 Apr 16;20(4):e0321659. doi: 10.1371/journal.pone.0321659 (PMC12002537; doi:10.1371/journal.pone.0321659)
Supplement: Attachment — Submitted filename: PONE-D-23-23260R2 Review.docx [file pone.0321659.s012.docx]

Thank you for the work put into the manuscript PONE-D-23-23260 titled “Drivers of rodent community structure in an urban national park, Kenya”.

However, here are some major corrections that needs to be effected in order to improve the manuscript.

The title is perfect because it captures the whole focus the study even though I see an argument around bioindicator in the manuscript which was not reflective in the manuscript. I will have also preferred if the Nairobi National Park, Kenya replaces Urban National Park, Kenya.

**Introduction**

Page 4, line 52-56: Please check the punctuations of the sentence and I think the sentence is too long, it should be broken into two.

**Materials and Methods**

Page 6, Line 101-102: I was just wondering the relevance of this statement to the overall study objective.

Page 6, line 110: Please un-italicise words that are not scientific names.

Page 7, line 126-132: The sentence is too long and also please recast it because it has grammatical issues.

Page 7, line 133: Please recast sentence because it has grammatical errors.

Page 8, line 139-144: What was the rationale and supporting documents behind choice of baits, trapping and marking methods, and identification procedures for reproducibility.

Page 8, line 150: Please put space after full stop.

**Results**

Page 9, line 174: Remove full stop.

Page 13, line 124: Please maintain the NNP style for the study area for consistency.

Page 13, line 245: Please correct sentence for grammatical errors.

Page 13, line 251-257: There is no reference to the table where result is being discussed.

Page 15, line 296-297: Please input significant values.

Page 16, line 301: Please input significant values.

**Discussion**

Page 26, line 427, 430: Please adjust to reference style.

Page 28, line 458-459: Please adjust to reference style.
