# Supplementary material for: Drivers of rodent community structure in an Urban National Park, Kenya
Source: PLoS One. 2025 Apr 16;20(4):e0321659. doi: 10.1371/journal.pone.0321659 (PMC12002537; doi:10.1371/journal.pone.0321659)
Supplement: Attachment — Submitted filename: RESPONSE TO REVIEWER COMMENTS_PLOS ONE_240927.docx [file pone.0321659.s013.docx]

**27^th^ September 2024**

**Waheed Ali Panhwar, Ph.D,
Academic Editor,
PLOS ONE.**

**Dear Waheed Ali Panhwar, Ph.D,**

**RE:** Response to reviewer comments on Manuscript Number: PONE-D-23-23260R2 entitled “Drivers of Rodent Community Structure in an Urban National Park, Kenya”.

We thank you for the prompt review of the above-mentioned manuscript. We are grateful to the reviewer for the comments, which have helped to improve the manuscript. Below please find a point-by-point response to each comment and suggestion raised by the reviewers in red highlight. We have also revised the same with track changes. We hope that this modified version will meet the expectations of the reviewer and the editorial team.

Sincerely,

On behalf of all authors,

Olivia Wesula Lwande, corresponding author,

Department of Clinical Microbiology, Virology, 901-85

SE, University of Umeå, Sweden

olivia.lwande@umu.se

**Response to reviewer comments**

We acknowledge the positive comments from Reviewers #1, 2, 3 and 5. We believe the valuable suggestions raised and addressed have contributed towards the improvement of the manuscript.

Please use the space provided to explain your answers to the questions above. You may also include additional comments for the author, including concerns about dual publication, research ethics, or publication ethics. (Please upload your review as an attachment if it exceeds 20,000 characters)

**Reviewer #4:** This manuscript provides novel research into the significance of ongoing monitoring of ecosystems which are disturbed by human development and activities, with results applicable to land managers. While the manuscript generally reads well, it could improve through the addition of some fine-scale detail and further explanation of the results and their implications. Furthermore, incorporating the key findings in relation to effects on rodent diversity and abundance into the concluding remarks will ensure that readers grasp the main findings of the study clearly. There are also formatting inconsistencies through the manuscript which require correction to enhance the readability of the paper. The referencing, both in text and in the reference list, still needs some attention to adhere to the PLOS ONE referencing guidelines.
We have now addressed the formatting inconsistencies throughout the manuscript in red, highlighting and reformatting all references according to the PLOS ONE journal guidelines. See track changes. In addition, we have clarified the key findings and implications in the concluding remarks.
Detailed comments can be found below.
- Appears there may be extra spaces in the text between words/after full stops e.g. Lines 35, 70, 99, 105, 111, 173, 203, 210, 251, 335 (space before the comma), 404.

Thank you for the correction. The extra spaces between words and full stops have been removed.
- Line 36: may read better as “were identified:”

Thank you. Noted and revised. See track changes.

- Lines 41, 46: instead of including that human disturbance had no effect on the two separate metrics, could have a sentence after stating what did have an effect on the metrics stating that human disturbance had no effect in both models.

Thank you, we have noted and revised the statement. See track changes.

- Line 48: add “measured” before “biotic and abiotic factors” to limit observations to those factors that were investigated in this study.

Thank you. Noted and revised. See track changes.

- Line 49: “a threat to the faunal diversity” – can the authors truly extrapolate their findings to all faunal diversity in the NNP with the same relationships they found for rodents occurring for all species?

Thank you for your valuable suggestion. We have looked into your comment and omitted this statement as it does not extrapolate to all faunal diversity for all species of rodents.

- Line 54: change the full stop after the citation to a comma.

Thank you. Noted and revised. See track changes.

- Line 59: what frequency of monitoring is required? Ongoing?

Thank you. We have added the frequency of monitoring which is ongoing (see track changes)
- Line 62: outline what “the choice” is referring to e.g. the choice of what?

Thank you. We refer to suitable bioindicator species.
- Line 64: remove the “a” before survival.

Thank you. Noted and revised. See track changes.

- Lines 66, 68, 84, 91: inconsistent formatting of “e.g.” (e.g e.g., and e,g.,) – ensure all are formatted the same throughout the text.

Thank you, we have now re/formatted abbreviation “e.g.” for consistency.
- Line 68: remove the “;” after “prey”.

Thank you, we have omitted “;” after “prey”.
- Lines 87, 89, 107: the citations have circular brackets instead of square brackets.

Thank you, all citations have been changed to square brackets.

- Line 92: is it better to say “park boundaries”?

Thank you. Noted and revised. See track changes.

- Line 98: start the sentence with “The”.

Thank you. We have started the sentence with “The”. See track changes.

- Line 104: add “a” before “decline”.

Thank you. Noted and added. See track changes.

- Line 110: “grass species, and scattered low-canopy” text shouldn’t be in italics.

Thank you, we have removed the italics. See track changes.
- Line113-114 and 224-225: consider keeping format the same in relation to the use of “and” in the list of animals within the two lots of brackets (e.g. either use “and” before the last animal or remove it).

Thank you we removed “and” as suggested. See track changes.
- Line 116: consider consolidating the figure 1 title e.g. map showing study location of NNP in relation to Kenya (left) and Africa (middle)…

Thank you, we have noted and revised.
- Line 126-129: consider re-writing sentence more concisely (e.g. Rodents were sampled using a line transect approach using a stratified random design…) or break-up into two sentences. The “for a given effort to sample rodents” component of the sentence doesn’t flow well.

Thank you, we have opted to break/down the sentence into two. See track changes.
- Line 129-130: this sentence is repetitive of the information provided in the sentence preceding it. Either remove this information from the first sentence which would make that sentence more concise, or remove this sentence.

Thank you, we have removed the sentence.
- Line 130: replace “it is sensitive” with “they are sensitive”.

Thank you, we noted that this statement had already been previously revised as suggested by the reviewer.

- Line 132: I think “a long” should be one word “along”.

Thank you, we have revised” a long” as one word.
- Line 133: insert “as” before “sites”. What is the distance from park boundaries that sampling occurred at?

Thank you, we have inserted “as” before “sites”. The distance from park boundaries was 100m.

- Line 136: what is the distance from edge boundaries that trapping occurred at?

The distance from edge boundaries that trapping occurred at was 50m.
- Line 140: sentence may benefit from adding “traps” after “relocating”.

Thank you for the comments we have now added “traps” after “relocating”. See track changes.
- Line 143: a space is needed after the full stop. Consider replacing “of importance” with “recorded”. May also benefit from including that the species were recorded here too.

Thank you for the suggestions. We have included space after full stop and replaced replacing “of importance” with “recorded” as well as included the recorded species.
- Line 146: add s to “shrub”.

Thank you for the comments we have now added s to “shrub”. See track changes.
- Line 148, 150: consider using lower case m for the units as you have used lower case letters for other measurement units e.g. cm.

Thank you, we have revised the format to included the right case for the abbreviation e.g. cm.
- Line 150: a space is needed after the full stop.

Thank you, we have added space after the full stop.
- Line 151: consider adding “possible” after “taxa”.

Thank you, we have added “possible” after “taxa”.

- Line 154, 156: keep formatting consistent when referring to “savannah, riverine, forest” – they appear as both capitals and lower case.

Thank you for the comment, we have now ensured that the formatting for “savannah, riverine, forest” –is consistent throughout the document. We used lower case. See track changes.
- Line 158, 248: the reference to figure 1 isn’t consistent (e.g. one is capitalised and one isn’t).

Thank you for the comment, we have edited the case as per you’re the reviewer’s suggestion. See track changes.
- Lines 164-167: the sentence doesn’t read properly with the inclusion of brackets around site names.

Thank you for the comment. We have opted to omit the content in brackets and correct the grammar for clarity,
- Line 167: consider re-wording “site named” to “sites” and then replace the “was” (line 168) to “were”.

Thank you for the comment, we have reworded “site named” to “sites” and replaced the “was” (line 168) to “were”.

- Line 168: remove “savanna, forest and riverine” as is repetitive information.

Thank you, we have removed the repeated information. See track changes.
- Line 174: remove the extra “ .”

Thank you, we have removed extra.
- Line 180, 186, 191: is there relevant citations can include here?

Thank you for your suggestion. We have looked into the reviewer comment and noted that we already included relevant citations.

- Line 217-222: sentence is too long and needs to either be broken up or re-written. Remove the repeated “the rodent species richness, abundance, and diversity” section.

Thank you for the comments. We have opted to broken down the sentence and removed redundant words.
- Line 231, 427, 430, 458: citation referenced incorrectly.

Thank you for the comment. We have now cited the references according to the PLOS ONE Journal guidelines.
- Line 232: consider writing “ the smallest AIC value”.

Thank you for the comment. We have written the smallest AIC value.
- Line 235: consider writing” because non-lethal traps were used for…”

Thank you for the comment. We have revised the statement as per the reviewer’s suggestion see track changes.
- Line 244: use the abbreviation for NNP.

Thank you for the comment. We have now used the abbreviation for NNP.

- Line 245: add a comma after “J”.

Thank you for the comment. We have added a comma after “J”.

- Line 246: add a full stop at the end of the sentence.

Thank you for the comment. a full stop at the end of the sentence.

- Line 249: consider adding “sites” to the end of the sentence.

Thank you for the comment. We have added “sites” to the end of the sentence.
- Line 254: consider writing “where only a single species was caught” for improved clarity.

Thank you for the comment. We have clarified the statement. See track changes.
- Line 255: need to add in the close bracket.

Thank you for the comment. We have added in the close bracket.
- Lines 292, 293: add a “0” in front of the decimal point.

Thank you for the comment. We have added a “0” in front of the decimal point.
- Line 294: remove the comma after 6.

Thank you for the comment. We have removed the comma after 6.
- Line 298, 312: should “per cent” read “percent”?

Thank you for the comment. We have written “per cent” as one word.
- Line 300: should it read “control sites”?

Thank you for the comment. Yes, it should read control sites.
- Line 305: the results may benefit from adding in if the difference was significant or not.

Thank you, we have noted that the level of significance is indicated clearly in Table 6.
- Line 306: add in “and” before “tree density”. Remove the capital from “Vegetation”.

Thank you, we have added in “and” before “tree density” and removed the capital from “Vegetation”.
- Line 308: it may be beneficial to define what you mean by dominant (e.g. was it based on a threshold of X% cover of a site?). This may be beneficial in the Methods.

Thank you for the comment. We have noted and revised in the methods section.
- Line 309, 310: inconsistent formatting of savannah – sometimes with a capital, other times without.

Thank you for the comment. We have now reformatted the savannah throughout the entire document. We decided to use lower case.
- Line 311: was there any statistical analyses conducted for these comparisons? Was there significant differences in dominant plant species composition across the vegetation types?

Thank you for the comment. All the statistics are included clearly in the results section and in the relevant Tables and figures.

Report these.
- Line 326: remove “a” before “savannah”.

Thank you, we have removed “a” before “savannah”.
- Line 334: add “a” before “dominant tree”.

Thank you, we have added “a” before “dominant tree”.
- Line 339-340: inconsistent formatting of sp (e.g. sp, sp and sp.).

We have now reformatted the sp throughout the entire document.
- Line 348: add “more” before “abundant”

Thank you, we have added “more” before “abundant”.
- Line 349, 358, 368: suggest just referring to the supplementary table as “S1 Table” or “S2 Table” – relevant for all references to supplementary tables.

Thank you, noted and revised.
- Table 5: should the “wet season cf. dry season” be all on one line?

Thank you, noted and revised.

- Tables 6, 7, 8, 9: just wondering why the metrics are in all capitals (and table 9 also bold).

Thank you for the comment. We have revised the tables 6, 7, 8 and 9 to have the same format.
- Line 370: space needed in “Table7”.

We have included space in “Table7”.
- Table 9: should the forest for the second and third lines all be on one line?

Thank you, noted and revised.

- Line 392: “in” should have a capital I.

We have capitalized I.
- Line 394: end bracket should not be in italics.

This was an oversight on our part, we have removed italics on the end bracket.
- Line 398: do the authors mean globally in reference to abundance or just within the study area?

Thank you for the comment. The authors refer to the tropics which we have added in the manuscript.
- Line 404: remove “a” before “significant”.

Thank you, we have removed “a” before “significant”.
- Line 405: this sentence is a very strong claim. I would suggest adding “can affect small mammal populations” as otherwise the sentence reads that this is always the case.

Thank you for the comment. We have noted and revised the statement as per your suggestion.
- Line 412: is there a citation for the distribution?

Noted, we have included the citation for the distribution.
- Line 415: space required in “H.kerbispeterhansi”.

Thank you we have added space in “H.kerbispeterhansi” as it is supposed to be written in scientific nomenclature. We have also italicized it.
- Line 418: is there any evidence to support this claim in this study?

Thank you for the comment. We have previously provided the evidence to support the study.
- Line 424: clarify whether this refers to in this study or in the aforementioned studies.

Thank you for the comment. We have clarified that this refers to the aforementioned studies. See track changes.
- Line 429: clarify whether this refers to native or introduced species or both.

Thank you for the comment. This refers to both native and introduced species.
- Line 453: perhaps a different word than “advances” would be better here.

Thank you, we have noted and revised. We have opted to use “promotes” instead of “advances”.
- Line 456: perhaps add analyses or similar to the end of the sentence. Also, remove the “s” from “types”.
- Line 457: could you provide examples of the different resources required?

Thank you for your comment, we have provided the different resources as required by the reviewer. See track changes.
- Line 458: authors could expand on what they mean by “influences” e.g. positive/negative etc.

We have opted not to use “influences” e.g. positive/negative etc but instead rephrase and use impacting considering high forest cover was linked to high rodent diversity with regards to species and vice-versa.

- Line 460: suggest re-writing this sentence to improve readability.

Thank you for your comment. We have re-written the sentence. See track changes.
- Line 461: remove the “(“ in front of the square bracket.

Thank you, we have now removed the “(“in front of the square bracket. See track changes.
- Line 465: perhaps add “species” after Mus.

Thank you, we have now added “species” after Mus. See track changes.
- Line 471: the concluding paragraph may benefit from re-stating how the vegetation types influenced rodent diversity.

Thank you for the valuable suggestion, we have now added the information on how vegetation types influenced rodent diversity on our study.
- Line 473: including the effect on abundance (e.g. increase/decrease) will help the reader finish the article with a clear understanding of how your measured metrics affected it. It may also be stronger take-home message to re-write the end of the sentence to something like “anthropogenic habitat disturbance has no direct influence”.

Thank you for your valuable suggestion. We agree with the reviewer’s comments and have re-written the take-home message.
- References: spaces are required between the year, volume number and page numbers for the journal articles. Journal names also aren’t abbreviated as per PLOS ONE guidelines.

We have noted your comment and reformatted all the references according to the PLOS ONE guidelines.
- Lines 534, 594, 601: have random numbers associated with them and no further information.

We have removed the random numbers.

- Lines 628-630: suggest not including “.pdf” in the table title names.

We have noted and removed “.pdf” in the table title names.

- Figure titles: keep consistent in formatting throughout the manuscript – some ended in full stops while others didn’t.

Thank you for the comment. We have now ensured that the formatting of all the figure titles is consistent throughout the manuscript.
