# Supplementary material for: Drivers of rodent community structure in an Urban National Park, Kenya
Source: PLoS One. 2025 Apr 16;20(4):e0321659. doi: 10.1371/journal.pone.0321659 (PMC12002537; doi:10.1371/journal.pone.0321659)
Supplement: Attachment — Submitted filename: A rebuttal letter.docx [file pone.0321659.s014.docx]

**Clement Ameh Yaro, Ph.D
Academic Editor
PLOS ONE**

**27^th^ January 2025**

**Dear Clement Ameh Yaro, Ph.D,**

**RE**: Response to reviewer comments on Manuscript Number: PONE-D-23-23260R3-Drivers of Rodent Community Structure in an Urban National Park, Kenya

We appreciate your time and work in ensuring we receive relevant feedback from all reviewers designated #5 and #6 on the aforementioned manuscript. The comments have helped us improve the manuscript. Please find a point-by-point response to each of the reviewers' comments and suggestions in red text below. We have also altered it with track adjustments. We hope the amended version meets the reviewers' and editorial team's expectations.

Thank you for your unwavering support.

Sincerely,

On behalf of all authors,

Olivia Wesula Lwande, corresponding author,

Department of Clinical Microbiology, Virology, 901-85

SE, University of Umeå, Sweden

olivia.lwande@umu.se

Journal Requirements:

Please review your reference list to ensure that it is complete and correct. If you have cited papers that have been retracted, please include the rationale for doing so in the manuscript text, or remove these references and replace them with relevant current references. Any changes to the reference list should be mentioned in the rebuttal letter that accompanies your revised manuscript. If you need to cite a retracted article, indicate the article’s retracted status in the References list and also include a citation and full reference for the retraction notice.

Thank you for your comment. We have reviewed all the cited references and the reference list to ensure it conforms to your esteemed journal PLOS ONE reference guidelines.


Reviewers' comments:

Reviewer's Responses to Questions

**Comments to the Author**

1. If the authors have adequately addressed your comments raised in a previous round of review and you feel that this manuscript is now acceptable for publication, you may indicate that here to bypass the “Comments to the Author” section, enter your conflict of interest statement in the “Confidential to Editor” section, and submit your "Accept" recommendation.

Reviewer #5: All comments have been addressed

Thank you for taking the time to review our manuscript. We are pleased to know that we have adequately addressed all the comments as per the reviewers' suggestions.

Reviewer #6: All comments have been addressed

We thank the reviewer for the time and effort invested in reviewing our manuscript. We are glad to know that we have managed to work on the comments raised satisfactorily.

2. Is the manuscript technically sound, and do the data support the conclusions?

The manuscript must describe a technically sound piece of scientific research with data that supports the conclusions. Experiments must have been conducted rigorously, with appropriate controls, replication, and sample sizes. The conclusions must be drawn appropriately based on the data presented.

Reviewer #5: Yes

Reviewer #6: Yes

We appreciate the positive comments from reviewers #5 and #6 concerning our manuscript being technically sound and appropriately drawing our conclusion based on the data demonstrated.

3. Has the statistical analysis been performed appropriately and rigorously?

Reviewer #5: Yes

Reviewer #6: Yes

Thank you for your positive response to the questions. We acknowledge that reviewers #5 and #6 have approved that the statistical analysis has been performed appropriately and rigorously.

4. Have the authors made all data underlying the findings in their manuscript fully available?

The [PLOS Data policy](https://eur01.safelinks.protection.outlook.com/?url=http%3A%2F%2Ftrack.editorialmanager.com%2FCL0%2Fhttp%3A%252F%252Fwww.plosone.org%252Fstatic%252Fpolicies.action%2523sharing%2F1%2F010f019489a88a2f-86be0664-6c32-4ac2-8197-fe9ace509710-000000%2FFY44l76wGwx4qrZSfKfZaG3p-URYK3G0IW1oBvhM7d0%3D194&data=05%7C02%7Colivia.lwande%40umu.se%7Cf95b4b66c0f248cead2308dd3aae77cd%7C5a4ba6f9f5314f329467398f19e69de4%7C0%7C0%7C638731243194877039%7CUnknown%7CTWFpbGZsb3d8eyJFbXB0eU1hcGkiOnRydWUsIlYiOiIwLjAuMDAwMCIsIlAiOiJXaW4zMiIsIkFOIjoiTWFpbCIsIldUIjoyfQ%3D%3D%7C0%7C%7C%7C&sdata=MADGdq8C6a5v0lVwKgl1vhToUI%2BkP7NI3%2FTczrM6B7g%3D&reserved=0) requires authors to make all data underlying the findings described in their manuscript fully available without restriction, with rare exception (please refer to the Data Availability Statement in the manuscript PDF file). The data should be provided as part of the manuscript or its supporting information, or deposited to a public repository. For example, in addition to summary statistics, the data points behind means, medians and variance measures should be available. If there are restrictions on publicly sharing data—e.g. participant privacy or use of data from a third party—those must be specified.

Reviewer #5: Yes

Reviewer #6: Yes

Thank you for the reply. We have provided all the data representing all the study findings. This has also been confirmed by reviewers #5 and #6.

5. Is the manuscript presented in an intelligible fashion and written in standard English?

PLOS ONE does not copyedit accepted manuscripts, so the language in submitted articles must be clear, correct, and unambiguous. Any typographical or grammatical errors should be corrected at revision, so please note any specific errors here.

Reviewer #5: Yes

Reviewer #6: Yes

Thank you for your positive reply. We agree with reviewers #5 and # 6 that the language and grammar used in the manuscript is in standard English. All the typographical and grammatical errors were adequately addressed during the later review process (version R3).

6. Review Comments to the Author

Reviewer #5: All authors have answered queries raised with regards to improving this manuscript. I want to say to the authors, Well done!

Thank you very much for the positive comment and for supporting our manuscript. We appreciate your dedication and scientific input in improving the manuscript.

Reviewer #6: The manuscript focuses on the rodent assemblage in a national park embedded within an urban matrix, making it highly relevant for understanding the wildlife in these areas, which are essential for biodiversity conservation. The manuscript is well-written and, from what I can see, has improved substantially since the initial version submitted.

Thank you for the encouraging comment. We acknowledge that indeed the manuscript has improved a lot due to your valuable input. We appreciate all your effort and hope that it will meet the expectations of the editorial team.

I only have two minor comments:

- I could not find information about the years and months when the rodent sampling was conducted. I suggest adding this information.

Thank you for the comment. Rodent sampling was conducted from December 2020 to June 2021 (see page 7 line 126) in the materials and methods section under the sub-heading entitled “Rodent community survey”.

- It would be helpful to include details about the size of the national park (in km²) for readers who may not be familiar with it.

The Nairobi National Park is 117 km² (see page 6 line 99 in the materials and methods under subheading entitled “study area”. This is supported by reference
“Owino AO, Kenana ML, Webala P, Andanje S, Omondi PO. Patterns of variation of herbivore assemblages at Nairobi National Park, Kenya, 1990-2008. Journal of Environmental Protection. 2011;02(1):855-866.”

7. PLOS authors have the option to publish the peer review history of their article ([what does this mean?](https://eur01.safelinks.protection.outlook.com/?url=https%3A%2F%2Ftrack.editorialmanager.com%2FCL0%2Fhttps%3A%252F%252Fjournals.plos.org%252Fplosone%252Fs%252Feditorial-and-peer-review-process%2523loc-peer-review-history%2F1%2F010f019489a88a2f-86be0664-6c32-4ac2-8197-fe9ace509710-000000%2FC8Rj58HJhKYQpuoI0FGFYxzp8Qi3insCl70tBAZXuts%3D194&data=05%7C02%7Colivia.lwande%40umu.se%7Cf95b4b66c0f248cead2308dd3aae77cd%7C5a4ba6f9f5314f329467398f19e69de4%7C0%7C0%7C638731243194885898%7CUnknown%7CTWFpbGZsb3d8eyJFbXB0eU1hcGkiOnRydWUsIlYiOiIwLjAuMDAwMCIsIlAiOiJXaW4zMiIsIkFOIjoiTWFpbCIsIldUIjoyfQ%3D%3D%7C0%7C%7C%7C&sdata=3P%2FNV9uKcEowOX%2FMIz5RLHSHD2dj%2BagiI%2FeYxQsbPYE%3D&reserved=0)). If published, this will include your full peer review and any attached files.

All authors agree to the option to publish the peer-review history of our article for transparency and accountability.

If you choose “no”, your identity will remain anonymous but your review may still be made public.


**Do you want your identity to be public for this peer review?** For information about this choice, including consent withdrawal, please see our [Privacy Policy](https://eur01.safelinks.protection.outlook.com/?url=https%3A%2F%2Ftrack.editorialmanager.com%2FCL0%2Fhttps%3A%252F%252Fwww.plos.org%252Fprivacy-policy%2F1%2F010f019489a88a2f-86be0664-6c32-4ac2-8197-fe9ace509710-000000%2FSyrEnU1eLP4cjEHmMft2r1RwODe-jiLEDq_YQdhcstM%3D194&data=05%7C02%7Colivia.lwande%40umu.se%7Cf95b4b66c0f248cead2308dd3aae77cd%7C5a4ba6f9f5314f329467398f19e69de4%7C0%7C0%7C638731243194894784%7CUnknown%7CTWFpbGZsb3d8eyJFbXB0eU1hcGkiOnRydWUsIlYiOiIwLjAuMDAwMCIsIlAiOiJXaW4zMiIsIkFOIjoiTWFpbCIsIldUIjoyfQ%3D%3D%7C0%7C%7C%7C&sdata=wSJrApZSC%2BDDmRkgexWCs8aCQ1P5qTmdXLv0SaZX%2FXk%3D&reserved=0).

Reviewer #5: No

Reviewer #6: No

We respect the reviewers' decision to remain anonymous.
